# Conduction system pacing compared with biventricular pacing for cardiac resynchronization therapy: a systematic review and meta-analysis

**DOI:** 10.3389/fcvm.2026.1776637

**Published:** 2026-06-05

**Authors:** Jing Guo, Weigang Luo, Sijia Zhao, Haoran Cui, Xia Wang, Xingjian Li

**Affiliations:** Department of Cardiology, Baoji Central Hospital, Baoji, Shaanxi, China

**Keywords:** biventricular pacing, cardiac resynchronization therapy, clinical outcomes, conduction system pacing, meta-analysis

## Abstract

**Background:**

Conduction system pacing (CSP) has emerged as a physiological alternative to biventricular pacing (BVP) for cardiac resynchronization therapy (CRT) in patients with heart failure (HF) with reduced ejection fraction (HFrEF). This systematic review and meta-analysis aimed to comprehensively compare the clinical efficacy and safety of these two strategies using the most up-to-date evidence.

**Methods:**

PubMed, Embase, Web of Science, and Cochrane Library were systematically searched up to March 2026 for randomized controlled trials (RCTs) and observational studies comparing CSP with BVP in adult HF patients (LVEF ≤ 50%). Primary outcomes included changes in LVEF, NYHA class, QRS duration, HF hospitalization (HFH), and all-cause mortality (ACM). Secondary outcomes included echocardiographic response, procedural parameters, and complications. Random-effects models were used. Heterogeneity was assessed using the I^2^ statistic. Publication bias was assessed using funnel plots, Egger's test, and trim-and-fill analysis. Certainty of evidence was appraised using the GRADE framework.

**Results:**

35 studies (10 RCTs, 25 observational; *N* = 7,019) were included. Compared with BVP, CSP was associated with greater improvement in LVEF (MD: 4.22%, 95%CI: 2.74%–5.70%; I^2^ = 72%), NYHA class (MD: −0.34, 95%CI: −0.47 to −0.21; I^2^ = 30%), and QRS narrowing (MD: −19.60 ms, 95%CI: −24.18 to −15.02 ms; I^2^ = 83%). CSP significantly reduced HFH risk (RR: 0.65, 95%CI: 0.49–0.87; I^2^ = 50%) and echocardiographic non-response (RR: 0.58, 95%CI: 0.41–0.82; I^2^ = 70%), while increasing super-response (RR: 1.86, 95%CI: 1.43–2.43; I^2^ = 34%). ACM was comparable between groups (RR: 0.87, 95%CI: 0.62–1.22). CSP was associated with shorter fluoroscopy time (MD: −5.04 min, 95%CI: −8.62 to −1.45 min), with similar complication rates. Benefits were most pronounced in patients with classical CRT indications (LVEF ≤ 35% with LBBB) and confirmed conduction system capture. Publication bias was detected for LVEF; trim-and-fill analysis confirmed directional benefit (adjusted MD: 2.14%). GRADE assessment demonstrated low to very low certainty of evidence.

**Conclusion:**

CSP may be associated with superior echocardiographic and electrocardiographic outcomes compared with BVP, but the overall certainty of the evidence remains low to very low. These findings should be considered hypothesis-generating and highlight the urgent need for large-scale, adequately powered RCTs to validate the potential benefits of CSP before its widespread adoption in routine clinical practice.

**Systematic Review Registration:**

https://www.crd.york.ac.uk/PROSPERO/view/CRD420251074973, identifier CRD420251074973.

## 1 Introduction

Cardiac resynchronization therapy (CRT) delivered via BVP remains a cornerstone in the management of patients with symptomatic heart failure (HF), a left ventricular ejection fraction (LVEF) below 35%, and a widened QRS complex, despite receiving optimal medical therapy, or in those requiring a high burden of ventricular pacing. Many clinical trials have demonstrated that BVP-based CRT effectively corrects left bundle branch block (LBBB), improves cardiac function, reverses left ventricular remodeling, and reduces both heart failure hospitalization (HFH) and all-cause mortality (ACM) (1–3). However, BVP-CRT is associated with several procedural and technical limitations, such as coronary sinus cannulation failure, lack of suitable target veins, phrenic nerve stimulation, elevated left ventricular pacing thresholds, and lead dislodgement (4, 5). Furthermore, nearly one-third of patients undergoing BVP fail to achieve significant clinical or echocardiographic improvement (6, 7). Therefore, there remains an ongoing need to refine and explore alternative pacing strategies.

Cardiac physiologic pacing (CPP), designed to restore or preserve ventricular synchrony, can be achieved either through conventional biventricular pacing (BVP-CRT) or via conduction system pacing (CSP), which directly engages the heart's native conduction pathways.

CSP comprises His bundle pacing (HBP) and left bundle branch area pacing (LBBAP). LBBAP includes two distinct subtypes: left bundle branch pacing (LBBP), characterized by definitive capture of the left bundle branch, and non-selective left ventricular septal pacing (LVSP), in which true left bundle branch capture (LBBP) is not achieved.

Both LBBAP and HBP deliver electrical stimulation within specialized conduction tissue to maintain synchronous electrical and mechanical ventricular activation (8). HBP, through direct engagement of the His bundle, preserves near-normal ventricular activation and is regarded as the most physiological form of cardiac pacing. Previous studies have shown that HBP can markedly improve cardiac function by correcting typical LBBB (9–12). Nonetheless, its wider clinical adoption has been constrained by technical challenges such as difficulty in precise lead placement, high capture thresholds, low sensing amplitudes, and a higher risk of loss of capture. In addition, HBP is less suitable for patients with distal or diffuse conduction system disease (13–16).

Both LBBP and LVSP can narrow the QRS complex and may partially correct LBBB, although the degree of electrical resynchronization achieved with LVSP is inferior to that of definitive conduction system capture (LBBP or HBP). In patients with distal or diffuse conduction system disease, LBBAP may bypass diseased conduction segments or partially restore conduction synchrony. Furthermore, LBBAP has demonstrated favorable pacing characteristics, including stable thresholds and low rates of capture failure, making it a promising and increasingly adopted alternative in clinical practice (17).

To date, the comparative efficacy of BVP and CSP in the context of CRT remains inconclusive. In 2023, Gin et al. published a meta-analysis that encompassed four randomized controlled trials (RCTs) and eleven observational studies, encompassing 1,211 patients (18). Their results suggested that CSP may provide comparable or even superior resynchronization compared to BVP, as evidenced by greater QRS narrowing, improved LVEF, and better New York Heart Association (NYHA) functional class. Moreover, both HBP and LBBAP demonstrated similar therapeutic benefits, although LBBAP was associated with lower pacing thresholds.

Most available evidence evaluating CSP in the context of CRT is derived from cohort studies, with large-scale RCTs still limited. To address these gaps and provide an updated synthesis, we conducted a comprehensive meta-analysis integrating the latest available data. Unlike the study by Gin et al., our analysis includes 35 studies, comprising 10 RCTs and 25 cohort studies, with a total of 7,019 participants. We aim to expand upon previous findings by incorporating a larger and more heterogeneous patient population, thereby providing an up-to-date and rigorous evaluation of the comparative clinical efficacy of CSP versus BVP in patients undergoing CRT.

## 2 Methods

### 2.1 Literature search

Our study followed the Preferred Reporting Items for Systematic Reviews and Meta-Analyses (PRISMA) 2020 statement (19) and was registered in the PROSPERO (CRD420251074973) before commencement. PubMed, Embase, Web of Science, as well as the Cochrane Library, were retrieved until March 2026 for English literature comparing the efficacy and/or safety of CSP versus BVP for CRT. The search strategy involved the following terms: “CSP,” “HBP,” “LBBP,” “LBBAP,” “BVP,” “pacing,” and “biventricular.” The strategy is detailed in Supplementary Figure S1. In addition, the reference lists of eligible studies were manually screened to identify further relevant publications. Two independent reviewers conducted the literature retrieval and screening processes, and any discrepancies were resolved through discussion and consensus.

### 2.2 Eligible study identification

Inclusion criteria were: (1) study design: RCTs or observational studies (prospective or retrospective cohorts) directly comparing CSP with BVP; (2) population: adult patients (≥ 18 years) with symptomatic HF (NYHA functional class II-IV) and LVEF ≤ 50%; (3) CRT indication: patients fulfilling contemporary guideline-recommended indications for CRT, including: (a) classic LBBB with LVEF ≤ 35%; (b) non-LBBB intraventricular conduction delay with LVEF ≤ 35% and prolonged QRS duration; or (c) atrioventricular (AV) block or high-grade AV block requiring ventricular pacing, with established or anticipated pacing-induced cardiomyopathy (i.e., LVEF deterioration); (4) intervention/comparator: *de novo* CRT implantation or device upgrade using either BVP or CSP; (5) outcomes: reporting of at least one clinically relevant endpoint, including LVEF, paced QRS duration, NYHA functional class, HFH, ACM, echocardiographic super-response, echocardiographic non-response, left ventricular end-diastolic diameter (LVEDD), pacing threshold, or R-wave amplitude; (6) data sufficiency: provision of adequate data to calculate mean difference (MD) or risk ratio (RR); and (7) duplicate publications: in cases of multiple reports derived from the same cohort, inclusion of the most recent publication with the largest sample size.

Exclusion criteria were: (1) absence of full texts; (2) lack of direct comparison between CSP and BVP; (3) insufficient data for quantitative synthesis; and (4) in instances of suspected duplicate publication from overlapping study populations, the earlier or smaller-sample report was excluded.

### 2.3 Data extraction

Two reviewers independently extracted the following data, with discrepancies resolved through discussion: first author, publication year, study period, study design, country, sample size, patient demographics (mean age, sex distribution, body mass index), follow-up duration, procedural and fluoroscopy times, and predefined efficacy and safety outcomes. As per the PROSPERO protocol, outcomes were categorized as primary efficacy, secondary efficacy, and safety/procedural endpoints. Primary efficacy endpoints included changes in LVEF (%), NYHA functional class, and QRS duration (ms), as well as HFH and ACM. Secondary efficacy endpoints included echocardiographic super-response (defined as an absolute increase in LVEF ≥ 20% from baseline or a follow-up LVEF ≥ 50%), echocardiographic non-response (defined as an absolute increase in LVEF < 5%), changes in left ventricular volumes [left ventricular end-diastolic volume (LVEDV) and left ventricular end-systolic volume (LVESV)] and dimensions [LVEDD and left ventricular end-systolic diameter (LVESD)], and left atrial diameter (LAD). A summary of endpoint definitions is presented in Supplementary Table S1. Safety and procedural endpoints included pacing threshold (left ventricular or His-bundle lead), R-wave amplitude, ventricular lead impedance, fluoroscopy time, total procedural duration, and procedure-related complications (e.g., infection, pneumothorax, lead dislodgement, and pericardial effusion) (20, 21). When essential information was unavailable, corresponding authors were contacted to obtain missing data. Studies for which critical data could not be retrieved were excluded from the quantitative synthesis of the corresponding outcomes.

### 2.4 Quality assessment and certainty of evidence

#### 2.4.1 Methodological quality assessment of individual studies

The quality of RCTs was assessed using the Cochrane Handbook for Systematic Reviews of Interventions 5.1.0, which evaluates random sequence generation, allocation concealment, participants and personnel blinding, outcome assessment blinding, incomplete outcome data, selective reporting, as well as other possible sources of bias (22). Each domain was categorized as low, high, or unclear risk of bias, with studies exhibiting a greater number of low-risk domains considered to have higher methodological quality. Two investigators independently assessed study quality, and disagreements were resolved through consensus. For observational studies, including prospective and retrospective cohorts, methodological quality was appraised via the Newcastle-Ottawa Scale (NOS) (23). Studies with NOS scores ≥ 6 (out of 9) were deemed to have high quality, while those scoring < 6 were considered to have low quality. Quality assessments were independently conducted by two reviewers, with any inconsistencies resolved by discussion.

#### 2.4.2 Certainty of evidence assessment

The Grading of Recommendations Assessment, Development and Evaluation (GRADE) framework (24) was applied to assess the certainty of evidence for each outcome. Evidence was rated as high, moderate, low, or very low based on predefined domains, including risk of bias, inconsistency (heterogeneity), indirectness, imprecision, and publication bias. Factors that could increase certainty, such as a large magnitude of effect, evidence of a dose-response gradient, or the presence of plausible residual confounding that would attenuate the observed effect, were also considered. This structured assessment informed interpretation of the findings and the strength of the conclusions.

### 2.5 Statistical analysis

Data synthesis was performed using Review Manager version 5.4 (Cochrane Collaboration, Oxford, UK). Continuous variables were analyzed using MDs, and dichotomous variables using RRs, both with 95% confidence intervals (CIs). All analyses were performed using a random-effects model (DerSimonian and Laird method) to estimate pooled effect sizes.

For studies including two or more eligible CSP intervention groups (e.g., HBP, LBBAP, LBBP, or LVSP) sharing a common BVP control group, the approach recommended by the Cochrane Handbook for Systematic Reviews of Interventions was applied to avoid unit-of-analysis errors. For dichotomous outcomes, the number of events and total sample size in the shared BVP control group were divided equally across the number of CSP intervention arms (*k*). For continuous outcomes, original means and standard deviations were retained for each split control subgroup. Each resulting pairwise comparison was treated as independent and pooled using a random-effects model.

Heterogeneity was assessed via the I^2^ statistic and the Cochrane Q test (25), with I^2^ > 50% or *P* < 0.10 indicating substantial heterogeneity. To explore potential sources of heterogeneity, prespecified subgroup analyses were conducted, and pooled effect estimates with 95% CIs were calculated within each subgroup. Between-subgroup differences were formally evaluated using interaction *P* values. Given inherent differences in study design, all primary and secondary outcomes were stratified by study type (RCTs versus observational studies).

For ACM and HFH, some studies reported hazard ratios (HRs) while others reported RRs. Because ACM and HFH are relatively infrequent events, HRs and RRs were considered approximately comparable measures of relative risk. For studies reporting HRs, the generic inverse-variance method was applied, pooling log-transformed HRs and corresponding standard errors utilizing a random-effects model. Subgroup analyses were further conducted according to effect measure type (HR versus RR), and formal tests for subgroup differences were carried out to evaluate potential variation in treatment effects.

The robustness of the pooled estimates was rated via sensitivity analyses using two approaches: (1) sequential exclusion of individual studies [leave-one-out (LOO) analysis], and (2) exclusion of studies requiring data transformation (e.g., conversion from medians or ranges).

Publication bias was visually assessed using funnel plots generated in Review Manager 5.4 and statistically evaluated via Egger's regression test (26) performed in Stata 18.0 (StataCorp, College Station, TX, USA), with *P* < 0.05 indicating statistical significance. When evidence of publication bias was detected (i.e., funnel plot asymmetry and a positive Egger's test), a Duval and Tweedie trim-and-fill analysis (27) was performed to estimate the potential impact of missing studies. This nonparametric method imputes hypothetical studies to restore funnel plot symmetry and recalculates the pooled effect estimate incorporating the imputed data. The bias-adjusted estimate was used to evaluate the robustness of the primary findings.

## 3 Results

### 3.1 Literature search and study characteristics

**Figure 1:**
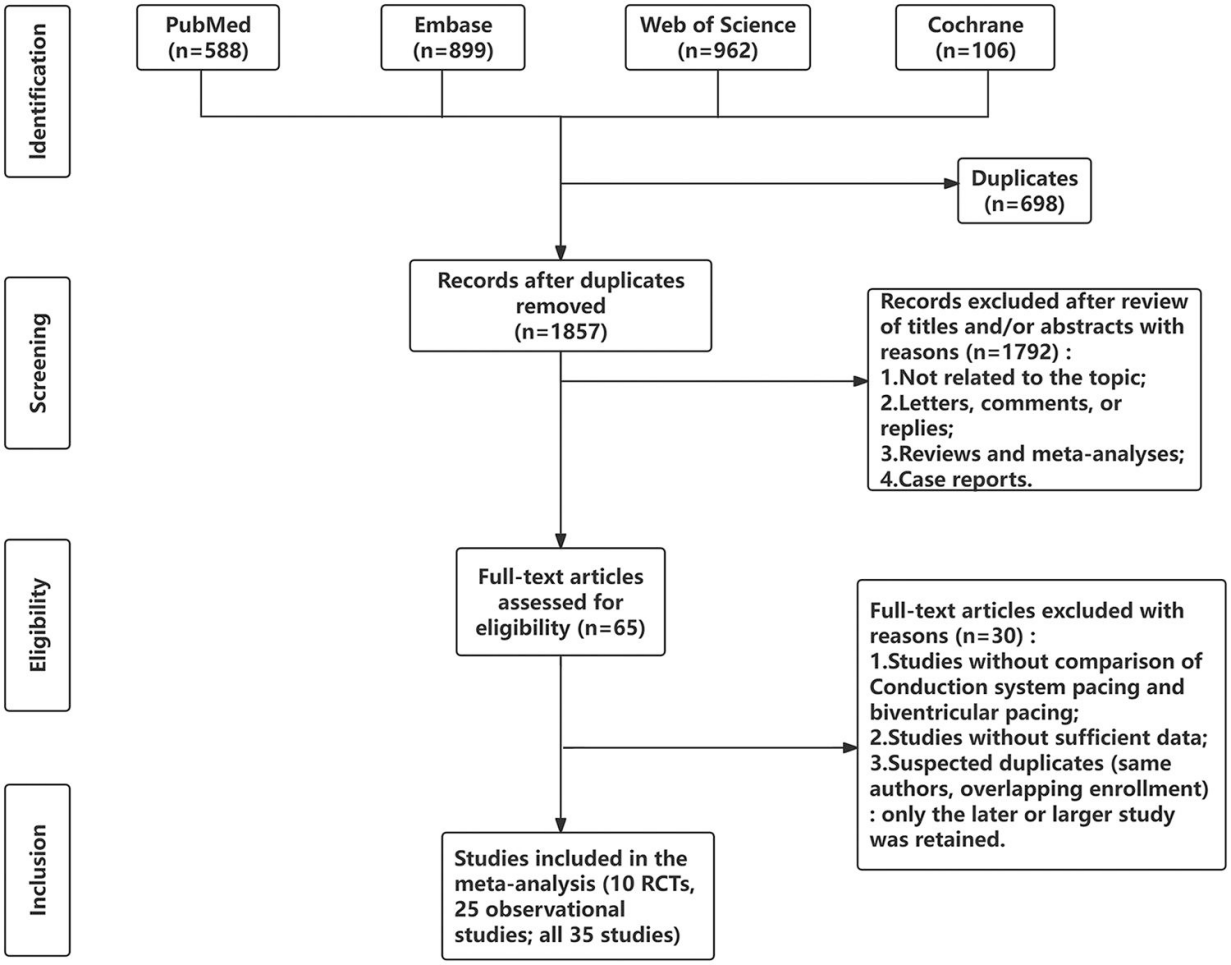
Flowchart of the systematic search and selection process.

Our search and selection process is presented in Figure 1. Two thousand five hundred and fifty-five relevant publications were initially identified across PubMed (*n* = 588), Embase (*n* = 899), Web of Science (*n* = 962), and Cochrane Library (*n* = 106). After duplicate removal and exclusion of irrelevant records, 35 full-text articles encompassing 35 studies with 39 comparative groups and 7,019 patients (CSP: 3,577; BVP: 3,442) were encompassed in the pooled analysis (28–62). Of these, 13 were prospective cohort studies (28–30, 32, 34, 37, 38, 45–47, 54, 57, 58), 12 were retrospective cohort studies (31, 33, 35, 39–42, 44, 48, 50, 52, 56), and 10 were RCTs (36, 43, 49, 51, 53, 55, 59–62). Table 1 displays the basic characteristics of the 39 comparative groups. The study quality assessment is provided in Supplementary Figure S2 and Supplementary Table S2.

**Table 1:** Basic characteristics of the 39 comparative groups.

References	Study period	Region	Study design	Patient Population Characteristics	Intervention	Control	Patients		Mean follow-up	Age	
							On	Off		On	Off
Chen et al. (28)	2018–2019	China	prospective cohort	CRT indication & LBBB	LBBAP-CRT	BVP	49	51	12	67.14 ± 8.88	64.37 ± 8.74
Chen et al. (29)	2019–2021	China	prospective cohort	CRT indication & IVCD	LOT-CRT	BVP	30	55	24	63.7 ± 13.3	63.5 ± 10.8
Chen et al. (30)	2019–2021	China	prospective cohort	CRT nonresponders	Upgrade LBBAP	BVP	44	44	22	64.73 ± 9.62	64.52 ± 11.95
Chen et al. (31)	2018–2022	China	retrospective cohort	CRT indication & NICM & low septal scar	LBBAP	BVP	30	43	33.7	58.23 ± 12.55	57.05 ± 11.71
Chen et al. (31)	2018–2022	China	retrospective cohort	CRT indication & NICM & high septal scar	LBBAP	BVP	21	53	33.7	56.76 ± 10.30	56.64 ± 11.98
Diaz et al. (32)	2020–2023	USA	prospective cohort	CRT indication & LBBB	LBBAP	BVP-1	141	122	13	69.2 ± 10.3	68.8 ± 11.8
Diaz et al. (32)	2020–2023	USA	prospective cohort	CRT indication & LBBB	LVSP	BVP-2	31	121	14	70.2 ± 11.9	68.8 ± 11.9
Ezzeddine et al. (33)	2017–2021	USA	retrospective cohort	CRT indication, LVEF ≤ 50% + anticipate RV pacing	CSP	BVP	119	119	8.9	69.1 ± 13.1	70.6 ± 11.9
Frandsen et al. (59)	2018–2020	Denmark	RCT	CRT indication & LBBB	HBP	BVP	19	31	63	63.2 ± 9.2	67.4 ± 9.1
Guo et al. (34)	2018–2019	China	prospective cohort	CRT indication & LBBB	LBBAP	BVP	21	21	14.3	66.1 ± 9.7	65.1 ± 7.5
Herweg et al. (35)	2018–2022	15 international centers	retrospective cohort	CRT indication	LBBAP	BVP	707	707	25.2	69.9 ± 12	69.0 ± 12
Huang et al. (36)	2019–2020	China	RCT	LVEF < 40% & atrioventricular nodal ablation in patients with persistent AF	HBP	BVP	21	21	9	63.6 ± 12.1	65.0 ± 8.2
Kato et al. (37)	2017–2019	Japan	prospective cohort	CRT indication & LBBB	HBP-CRT	BVP	9	5	12	70.9 ± 14.9	71.0 ± 5.2
Li et al. (38)	2018	China	prospective cohort	CRT indication & LBBB	LBBAP	BVP	27	54	6	56.8 ± 10.1	NA
Liang et al. (39)	2018–2021	China	retrospective cohort	CRT indication, High to third-degree AVB	LBBAP	BVP	154	337	31	67.0 ± 8.98	62.30 ± 10.42
Ma et al. (40)	2017–2019	China	retrospective cohort	Bradyarrhythmias, AF and HFrEF	HBP	BVP	37	15	18.12	70.32 ± 12.36	66.80 ± 7.26
Ma et al. (41)	2017–2021	China	retrospective cohort	PICM	CSP(HBP/LBBAP)	BVP	37	11	27.78	65.69 ± 14.85	59.86 ± 10.79
Moriña-Vázquez et al. (42)	2018–2020	Spain	retrospective cohort	CRT indication & LBBB & NICM	HBP	BVP	52	51	12	66.83 ± 10.67	67.65 ± 9.92
Pujol-Lopez et al. (43)	2019–2021	Spain	RCT	CRT indication,AVB	CSP	BVP	35	35	6	65.7 ± 9.0	68.1 ± 9.0
Pujol-López et al. (44)	2019–2020	Spain	retrospective cohort	LVEF ≤ 5%,AVB	CSP	BVP	25	25	6	72 ± 9	69 ± 8
Pujol-Lopez et al. (60)	2019–2023	Spain	RCT	LVEF ≤ 35%, AVB	CSP(LBBP/HBP)	BVP	67	67	12	69 ± 9	69 ± 9
Senes et al. (45)	2019	Italy	prospective cohort	CRT indication,atrioventricular nodal ablation in patients with persistent AF	HBP/HBP + LV	BVP	27	27	9.7	76 ± 7	77 ± 7
Shroff et al. (46)	2019–2022	Australia	prospective cohort	CRT indication	LBBAP	BVP	51	50	33.7	70.2 ± 12.2	71.9 ± 8.05
Tan et al. (47)	2012–2018	Singapore	prospective cohort	CRT indication,High to third-degree AVB	CSP	BVP	48	48	19	70 ± 10	70 ± 12
Tang et al. (48)	2003–2021	USA	retrospective cohort	LVEF from > 35 to 50%	CSP(HBP/LBBAP)	BVP	16	20	42.7	74.9 ± 16.8	71.1 ± 12.8
Upadhyay et al. (49)	2016–2018	USA	RCT	CRT indication	HBP	BVP	16	24	12.2	63.4 ± 13.3	65.5 ± 12.2
Vijayaraman et al. (50)	2018–2022	15 international centers	retrospective cohort	CRT indication & LBBB	LBBAP	BVP	447	626	33	67 ± 12	68 ± 12
Vijayaraman et al. (51)	2021–2022	USA	RCT	CRT indication & LBBB	CSP(HBP/LBBAP/LBBAP + CS)	BVP	31	31	6	67.3 ± 14.5	66.8 ± 12.3
Vijayaraman et al. (52)	2018–2023	16 international centers	retrospective cohort	LVEF from > 35 to 50%	CSP(HBP/LBBAP)	BVP	826	178	29.5	73 ± 13	73 ± 12
Vinther et al. (53)	2018–2022	Denmark	RCT	CRT indication & LBBB	HBP	BVP	19	31	6	63.2 ± 9.2	67.4 ± 4.4
Wang et al. (54)	2019	China	prospective cohort	CRT indication & LBBB	LBBAP	BVP	10	30	6	64.8 ± 7.25	62.93 ± 10.33
Wang et al. (55)	2019–2021	China	RCT	CRT indication & LBBB	LBBAP	BVP	20	20	6	62.3 ± 11.2	65.3 ± 10.6
Wang et al. (56)	2018–2022	China	retrospective cohort	CRT indication & LBBB	LBBAP	BVP	85	45	28	65.6 ± 9.8	65.6 ± 10.5
Wu et al. (57)	2012–2018	China	prospective cohort	CRT indication & LBBB	HBP	BVP-1	49	27	12	68.3 ± 10	68.3 ± 10
Wu et al. (57)	2012–2018	China	prospective cohort	CRT indication & LBBB	LBBAP	BV-2	32	27	12	67.2 ± 13	68.3 ± 10
Zhu et al. (58)	2019–2020	China	prospective cohort	CRT indication	LBBAP	BVP	68	77	28.8	62.1 ± 12.4	63.6 ± 10.5
Zhu et al. (58)	2019–2020	China	prospective cohort	CRT indication	LVSP	BVP	38	76	29.8	62.8 ± 11.5	63.6 ± 10.5
Zimerman et al. (61)	2022–2023	Brazil	RCT	CRT indication & LBBB	CSP(LBBAP/HBP/LVSP)	BVP	87	86	12	61.0 ± 8.9	63.0 ± 9.6
Žižek et al. (62)	2022–2024	Slovenia	RCT	CRT indication & LBBB	LBBAP	BVP	31	31	22.1	65 ± 9.6	70 ± 14.8

### 3.2 Change in LVEF

**Figure 2:**
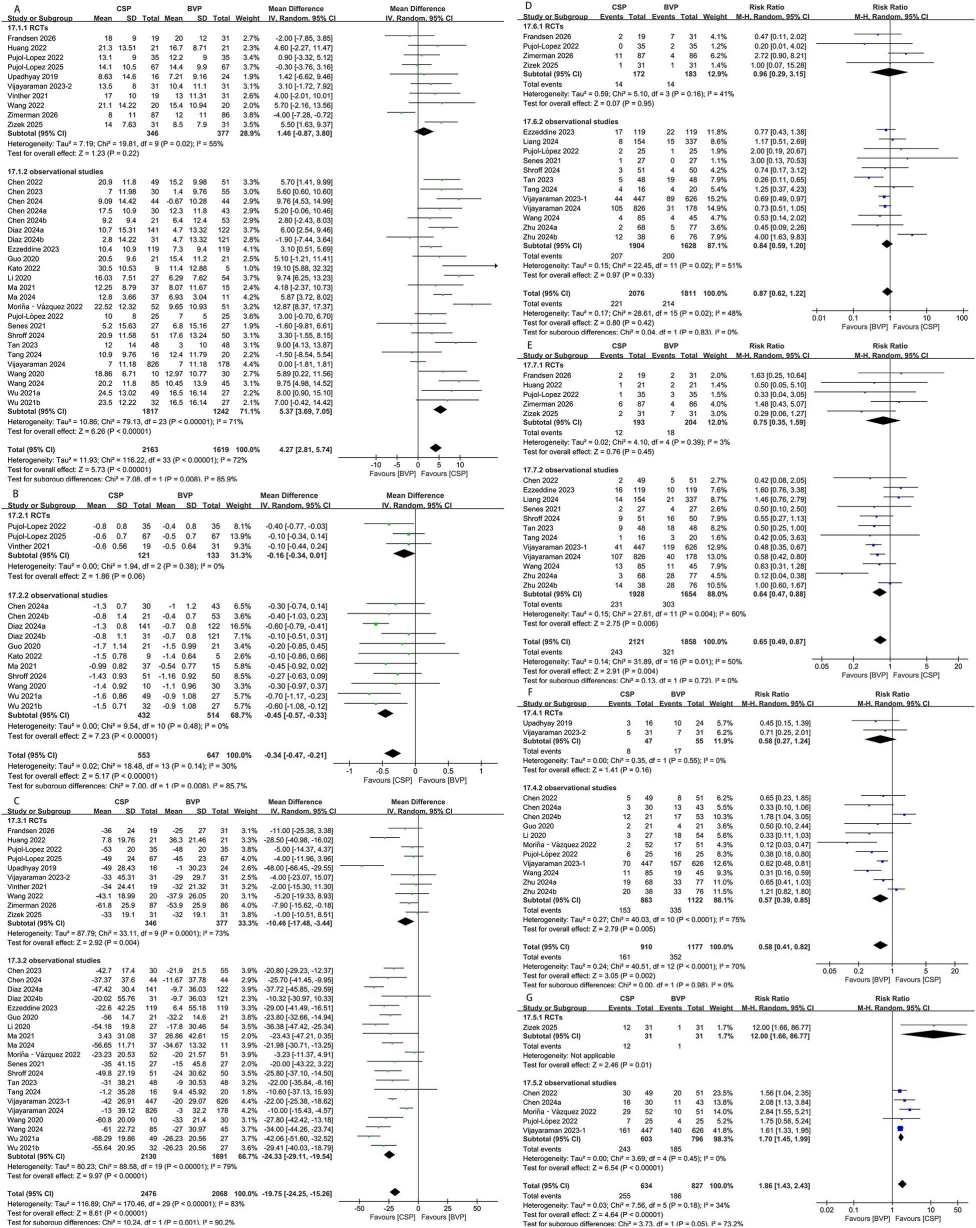
Forest plots of outcomes: **(A)** change in LVEF, **(B)** change in NYHA functional class, **(C)** change in QRS duration, **(D)** ACM, **(E)** HFH, **(F)** echocardiographic non-response, and **(G)** echocardiographic super-response.

34 comparative groups comprising 3,782 patients (CSP: 2,163; BVP: 1,619) reported LVEF change (28–34, 36–38, 40–49, 51–57, 59–62). Pooled analysis demonstrated that CSP was associated with a significantly greater improvement in LVEF compared with BVP (MD: 4.22%; 95% CI: 2.74%–5.70%; *P* < 0.00001), with substantial heterogeneity across studies (I^2^ = 72%, *P* < 0.00001; Figure 2A).

#### 3.2.1 Subgroup analyses

##### 3.2.1.1 Study design

Treatment effects differed significantly according to study design (*P* for interaction = 0.04). Among ten RCTs (723 patients), CSP demonstrated a trend toward greater LVEF improvement that did not reach statistical significance (MD: 1.46%; 95% CI: −0.87%–3.80%; *P* = 0.22; I^2^ = 55%). In contrast, 24 observational studies (3,059 patients) showed a significant benefit favoring CSP (MD: 5.37%; 95% CI: 3.69%–7.05%; *P* < 0.00001), although heterogeneity was substantial (I^2^ = 71%).

##### 3.2.1.2 CSP subtype

Most CSP subtypes were associated with significant LVEF improvement, including LBBAP (MD: 6.28%; 95% CI: 4.38%–8.18%; *P* < 0.00001; I^2^ = 25%), LBBP (MD: 6.48%; 95% CI: 4.41%–8.55%; *P* < 0.00001; I^2^ = 0%), HBP (MD: 5.13%; 95% CI: 1.04%–9.21%; *P* = 0.01; I^2^ = 69%), and mixed CSP strategies (MD: 1.77%; 95% CI: −0.51%–4.06%; *P* = 0.13; I^2^ = 79%). In contrast, LVSP did not demonstrate a significant benefit (MD: −1.90%; 95% CI: −7.44%–3.64%; *P* = 0.50; single study). Inclusion of LVSP contributed substantially to overall heterogeneity. The subgroup difference was significant when LVSP was included (*P* for interaction = 0.0002) but was attenuated after its exclusion (*P* for interaction = 0.01).

##### 3.2.1.3 Patient phenotype

Significant LVEF improvement was observed both in patients meeting traditional CRT criteria (LVEF ≤ 35% with LBBB; MD: 4.76%; 95% CI: 1.71%–7.81%; *P* = 0.002; I^2^ = 81%) and in those with alternative indications (MD: 3.88%; 95% CI: 2.29%–5.48%; *P* < 0.00001; I^2^ = 62%). No statistically significant difference was detected between these subgroups (*P* for interaction = 0.62; Table 2).

**Table 2:** Subgroup analysis of change in LVEF, change in NYHA functional class, and change in QRS duration.

Subgroup	Change in LVEF, %					Change in NYHA functional class					Change in QRS duration, ms				
	Study	MD [95%CI]	*P* value	I^2^	*P* for interaction	Study	MD [95%CI]	*P* value	I^2^	*P* for interaction	Study	MD [95%CI]	*P* value	I^2^	*P* for interaction
Total	34	4.22 [2.74,5.70]	< 0.00001	72%		14	−0.34 [−0.47, −0.21]	< 0.00001	30%		30	−19.60 [−24.18, −15.02]	< 0.00001	83%	
Study design															
RCT	10	1.46 [−0.87, 3.80]	0.22	55%	0.008	3	−0.16 [−0.34, 0.01]	0.06	0%	0.008	10	−10.46 [−17.48, −3.44]	0.004	73%	0.001
Observational study	24	5.37 [3.69, 7.05]	< 0.00001	71%		11	−0.45 [−0.57, −0.33]	< 0.00001	0%		20	−24.33 [−29.11, −19.54]	< 0.00001	79%	
CSP subtype															
LBBAP	8	6.28 [4.38, 8.18]	< 0.00001	25%	0.002 (including LVSP)/0.01 (excluding LVSP)	3	−0.30 [−0.56, −0.04]	0.02	0%	0.11 (including LVSP)/0.13 (excluding LVSP)	7	−23.53 [−31.06, −15.99]	< 0.00001	81%	0.06 (including LVSP)/0.04 (excluding LVSP)
LBBP	6	6.48 [4.41, 8.55]	< 0.00001	0%		4	−0.55 [−0.72, −0.39]	< 0.00001	0%		5	−25.22 [−35.14, −15.29]	< 0.00001	76%	
HBP	9	5.13 [1.04, 9.21]	0.01	69%		4	−0.34 [−0.64, −0.04]	0.03	36%		8	−21.95 [−35.49, −8.41]	0.001	88%	
LVSP	1	−1.90 [−7.44, 3.64]	0.5	/		1	−0.10 [−0.51, 0.31]	0.63	/		1	−10.32 [−30.97, 10.33]	0.33	/	
Mixed CSP Subtypes	10	1.77 [−0.51, 4.06]	0.13	79%		2	−0.21 [−0.50, 0.07]	0.14	43%		9	−11.97 [−17.93, −6.0]	< 0.0001	68%	
Patient Phenotype															
LVEF ≤ 35%&LBBB	13	4.76 [1.71, 7.81]	0.002	81%	0.62	5	−0.27 [−0.56, 0.02]	0.07	60%	0.77	12	−15.74[−23.34, −8.13]	< 0.00001	88%	0.19
Others	21	3.88 [2.29, 5.48]	< 0.00001	62%		9	−0.32 [−0.45, −0.18]	< 0.00001	0%		18	−22.32 [−28.40, −16.24]	< 0.00001	81%	

#### 3.2.2 Sensitivity analysis

**Figure 3:**
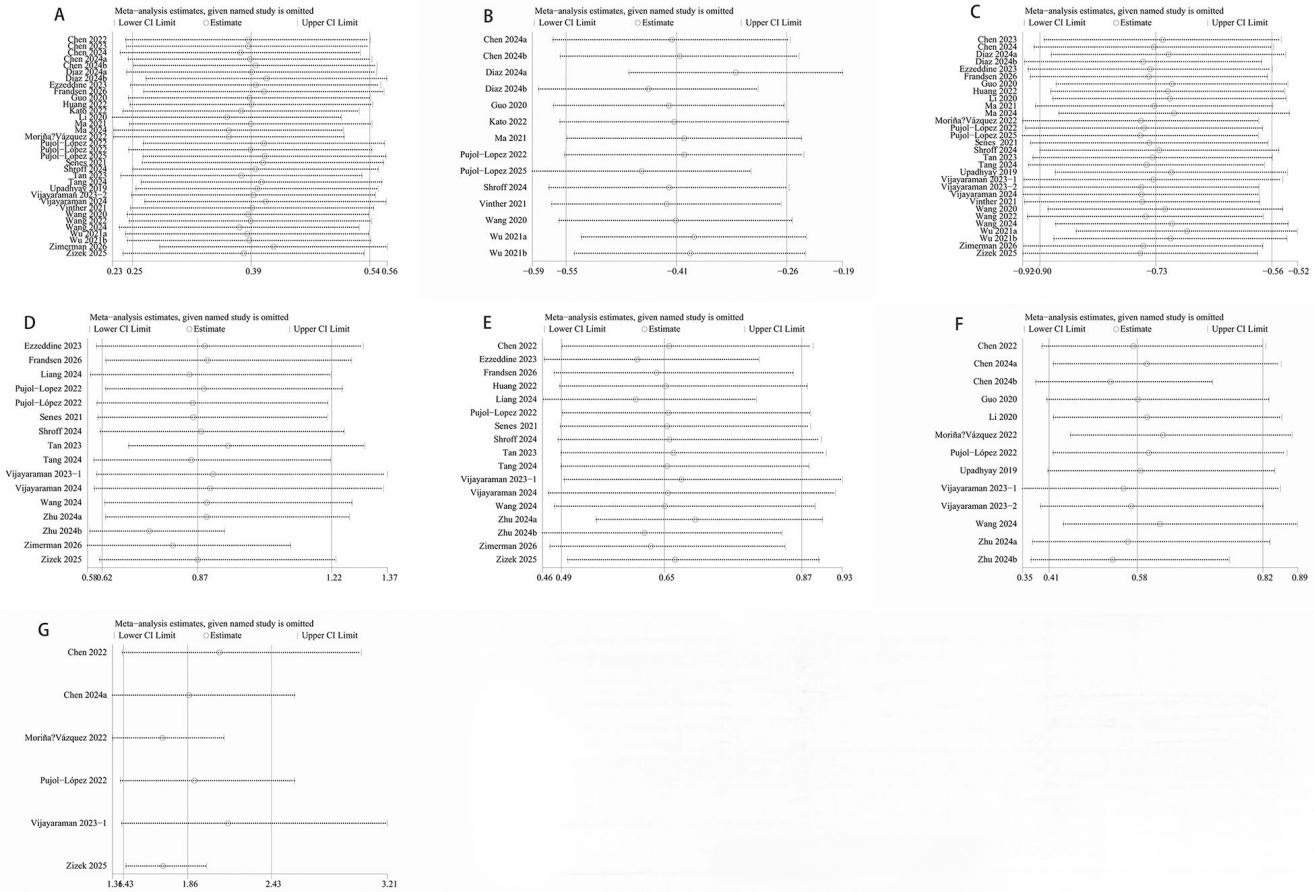
Sensitivity analysis: **(A)** change in LVEF, **(B)** change in NYHA functional class, **(C)** change in QRS duration, **(D)** ACM, **(E)** HFH, **(F)** echocardiographic non-response, and **(G)** echocardiographic super-response.

LOO analysis demonstrated that no individual study exerted a disproportionate influence on the pooled effect estimate (Figure 3A). After excluding four studies (Supplementary Table S3) that required data transformation, the pooled estimate from the remaining 30 studies (CSP: 2,037; BVP: 1,459) remained consistent with the primary analysis (MD: 3.70%; 95% CI: 2.26%–5.13%; *P* < 0.00001; I^2^ = 66%; Supplementary Figure S3A), indicating that data conversion did not materially affect the overall conclusions.

#### 3.2.3 Publication bias

**Figure 4:**
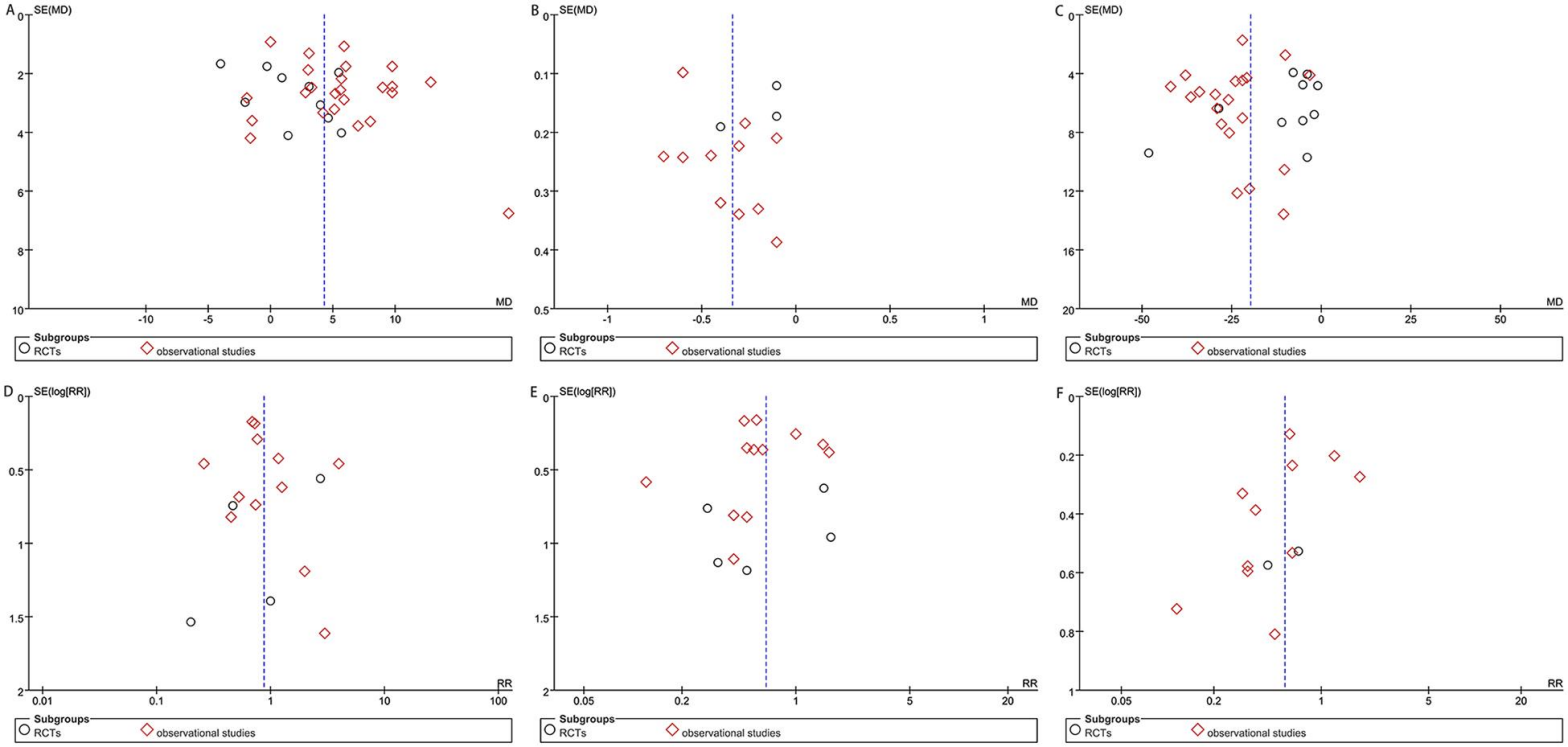
Funnel plots: **(A)** change in LVEF, **(B)** change in NYHA functional class, **(C)** change in QRS duration, **(D)** ACM, **(E)** HFH, **(F)** echocardiographic non-response.

Visual inspection of the funnel plot suggested asymmetry (Figure 4A), and Egger's test confirmed significant publication bias (*P* = 0.011). Accordingly, a trim-and-fill analysis was performed to account for potentially missing studies. After imputation, the adjusted pooled effect size was attenuated but remained statistically significant (MD: 2.14%; 95% CI: 1.92%–2.35%; Supplementary Figure S4), compared with the original estimate (MD: 4.22%; 95% CI: 2.74%–5.70%). Therefore, although publication bias may have inflated the magnitude of the observed treatment effect, the directional benefit of CSP in improving LVEF remained robust after correction.

### 3.3 Change in NYHA functional class

14 comparative groups comprising 1,197 patients (CSP: 553; BVP: 644) reported changes in NYHA functional class (30, 32, 34, 37, 40, 43, 46, 53, 54, 57, 60). The pooled analysis demonstrated that CSP was associated with a significantly greater improvement in NYHA class compared with BVP (MD: −0.34; 95% CI: −0.47–−0.21; *P* < 0.00001), with low between-study heterogeneity (I^2^ = 30%; Figure 2B).

#### 3.3.1 Subgroup analyses

##### 3.3.1.1 Study design

Treatment effects differed significantly according to study design (*P* for interaction = 0.008). Among three RCTs (254 patients), CSP showed a trend toward improved NYHA class that did not reach statistical significance (MD: −0.16; 95% CI: −0.34–0.01; *P* = 0.06; I^2^ = 0%). In contrast, eleven observational studies (946 patients) demonstrated a significant benefit favoring CSP (MD: −0.45; 95% CI: −0.57–−0.33; *P* < 0.00001; I^2^ = 0%).

##### 3.3.1.2 CSP subtype

Most CSP subtypes were associated with significant improvements in NYHA class, including LBBAP (MD: −0.30; 95% CI: −0.56–−0.04; *P* = 0.02; I^2^ = 0%), LBBP (MD: −0.55; 95% CI: −0.72–−0.39; *P* < 0.00001; I^2^ = 0%), HBP (MD: −0.34; 95% CI: −0.64–−0.04; *P* = 0.03; I^2^ = 36%), and mixed CSP (MD: −0.21; 95% CI: −0.50–0.07; *P* = 0.14). However, LVSP did not demonstrate a significant benefit (MD: −0.10; 95% CI: −0.51–0.31; *P* = 0.63; single study). Tests for subgroup differences indicated insignificant heterogeneity among CSP subtypes, irrespective of whether LVSP was included (*P* for interaction = 0.11 with LVSP included; *P* = 0.13 after excluding LVSP).

##### 3.3.1.3 Patient phenotype

Among patients meeting traditional CRT criteria (LVEF ≤ 35% with LBBB), a trend toward improved NYHA class was observed (MD: −0.27; 95% CI: −0.56–0.02; *P* = 0.07; I^2^ = 60%). In patients with other indications, a significant benefit was demonstrated (MD: −0.32; 95% CI: −0.45–−0.18; *P* < 0.00001; I^2^ = 0%). No significant difference was detected between these subgroups (*P* for interaction = 0.77; Table 2).

#### 3.3.2 Sensitivity analysis

LOO analysis confirmed the robustness of the pooled estimate, as exclusion of any single study did not materially alter the overall effect size (Figure 3B).

#### 3.3.3 Publication bias

Visual inspection of the funnel plot suggested slight asymmetry (Figure 4B). However, Egger's test did not detect significant publication bias (*P* = 0.232), supporting the robustness of the findings for NYHA functional class.

### 3.4 Change in QRS duration

30 comparative groups comprising 4,544 patients (CSP: 2,476; BVP: 2,068) reported QRS duration change (29, 30, 32–34, 36, 38, 40–43, 45–47, 59–62). The pooled analysis demonstrated that CSP was associated with a significantly greater shortening of QRS duration compared with BVP (MD:−19.60 ms; 95% CI: −24.18–−15.02 ms; *P* < 0.00001), with substantial between-study heterogeneity (I^2^ = 83%, *P* < 0.00001; Figure 2C).

#### 3.4.1 Subgroup analyses

##### 3.4.1.1 Study design

Treatment effects differed significantly according to study design (*P* for interaction = 0.001). Among ten RCTs (723 patients), CSP significantly reduced QRS duration (MD: −10.46 ms; 95% CI: −17.48–−3.44 ms; *P* = 0.004; I^2^ = 73%). Similarly, twenty observational studies (3,821 patients) demonstrated a significant benefit favoring CSP (MD: −24.33 ms; 95% CI: −29.11–−19.54 ms; *P* < 0.00001; I^2^ = 79%).

##### 3.4.1.2 CSP subtype

Most CSP subtypes were associated with significant QRS shortening, including LBBAP (MD: −23.53 ms; 95% CI: −31.06–−15.99 ms; *P* < 0.00001; I^2^ = 81%), LBBP (MD: −25.22 ms; 95% CI: −35.14–−15.29 ms; *P* < 0.00001; I^2^ = 76%), HBP (MD: −21.95 ms; 95% CI: −35.49–−8.41 ms; *P* = 0.001; I^2^ = 88%), and mixed CSP (MD: −11.97 ms; 95% CI: −17.93–−6.0 ms; *P* < 0.0001; I^2^ = 68%). In contrast, LVSP did not demonstrate a significant effect (MD: −10.32 ms; 95% CI: −30.97–10.33 ms; *P* = 0.33; single study). Tests for subgroup differences showed significant heterogeneity across CSP subtypes regardless of LVSP inclusion (*P* for interaction = 0.06 with LVSP included; *P* = 0.04 after excluding LVSP).

##### 3.4.1.3 Patient phenotype

Significant QRS shortening was observed both in patients meeting traditional CRT criteria (LVEF ≤ 35% with LBBB; MD: −15.74 ms; 95% CI: −23.34–−8.13 ms; *P* < 0.00001; I^2^ = 88%) and in those with other indications (MD: −22.32 ms; 95% CI: −28.40–−16.24 ms; *P* < 0.00001; I^2^ = 81%), with insignificant interaction between subgroups (*P* for interaction = 0.19; Table 2).

#### 3.4.2 Sensitivity analysis

LOO analysis confirmed the robustness of the pooled estimate, as no individual study exerted a disproportionate influence on the overall effect (Figure 3C). Additionally, after excluding five studies (Supplementary Table S3) that required data conversion, the pooled estimate from the remaining 25 studies remained consistent with the primary analysis (MD: −19.87 ms; 95% CI: −24.44–−15.29 ms; *P* < 0.00001; I^2^ = 80%; Supplementary Figure S3B), with substantially overlapping CIs. These findings indicate that data conversion did not materially affect the conclusions.

#### 3.4.3 Publication bias

Visual inspection of the funnel plot suggested slight asymmetry (Figure 4C), but Egger's test revealed insignificant publication bias (*P* = 0.278).

### 3.5 ACM

16 comparison groups (enrolling 3,887 patients, with 2,076 in the CSP group and 1,811 in the BVP group) reported ACM (33, 39, 43–48, 50, 51, 56, 58, 59, 61, 62). Pooled analysis of RRs demonstrated that ACM was comparable between the CSP and BVP groups (RR: 0.87; 95% CI: 0.62–1.22; *P* = 0.42), with moderate between-study heterogeneity (I^2^ = 48%, *P* = 0.02; Figure 2D).

**Figure 5:**
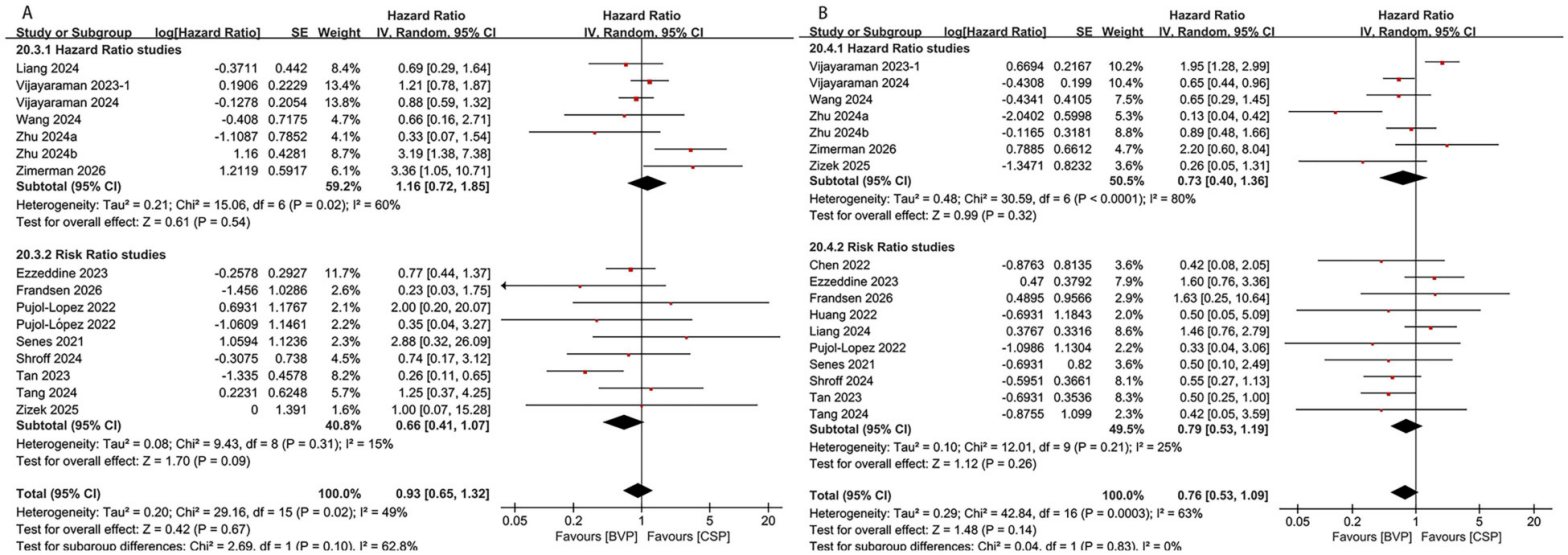
Forest plot of subgroup analysis stratified by effect measure type for **(A)** ACM and **(B)** HFH.

Among these studies, seven reported hazard ratios (HRs), and nine reported RRs. Given that ACM was a relatively rare event (incidence < 10% in most included studies), HRs and RRs were considered comparable, permitting subgroup analysis according to effect measure type. Pooled analysis of HRs showed an insignificant difference between CSP and BVP (HR: 1.16; 95% CI: 0.72–1.85; *P* = 0.54; I^2^ = 60%). Similarly, pooled analysis of RRs revealed an insignificant difference (RR: 0.66; 95% CI: 0.41–1.07; *P* = 0.09; I^2^ = 15%). A significant interaction was observed between subgroups stratified by effect measure type (*P* for interaction = 0.10; Figure 5A).

#### 3.5.1 Subgroup analyses

##### 3.5.1.1 Study design

No significant difference between RCTs and observational studies regarding treatment effect (*P* for interaction = 0.83). Four RCT (355 patients) showed an insignificant difference (RR: 0.96; 95% CI: 0.29–3.15; *P* = 0.95; I^2^ = 41%). Likewise, twelve observational studies (3,532 patients) demonstrated an insignificant difference (RR: 0.84; 95% CI: 0.59–1.20; *P* = 0.33; I^2^ = 51%).

##### 3.5.1.2 CSP subtype

LBBAP was associated with a significantly lower ACM risk (RR: 0.74; 95% CI: 0.55–0.99; *P* = 0.04; I^2^ = 0%), and mixed CSP demonstrated a trend toward benefit (RR: 0.80; 95% CI: 0.48–1.33; *P* = 0.38; I^2^ = 52%). In contrast, LVSP was associated with a significantly higher ACM risk (RR: 4.00; 95% CI: 1.63–9.83; *P* = 0.003). A significant difference across CSP subtypes was observed when LVSP was included (*P* for interaction = 0.01), which disappeared after excluding LVSP (*P* for interaction = 0.93), suggesting that heterogeneity was primarily driven by LVSP studies.

##### 3.5.1.3 Patient phenotype

A significant benefit was observed in patients meeting traditional CRT criteria (RR: 0.94; 95% CI: 0.43–2.05; *P* = 0.88; I^2^ = 49%). In contrast, an insignificant difference was observed among those with other indications (RR: 0.86; 95% CI: 0.56–1.33; *P* = 0.49; I^2^ = 51%), with insignificant interaction between subgroups (*P* for interaction = 0.84; Table 3).

**Table 3:** Subgroup analysis of ACM, HFH, echocardiographic non-response, and echocardiographic super-response.

Subgroup	ACM					HFH				
	Study	RR [95%CI]	*P* value	I^2^	*P* for interaction	Study	RR [95%CI]	*P* value	I^2^	*P* for interaction
Total	16	0.87 [0.62,1.22]	0.42	48%		17	0.65 [0.49, 0.87]	0.004	50%	
Study design										
RCT	4	0.96 [0.29, 3.15]	0.95	41%	0.83	5	0.75 [0.35, 1.59]	0.45	3%	0.72
observational study	12	0.84 [0.59, 1.20]	0.33	51%		12	0.64 [0.47, 0.88]	0.006	60%	
CSP subtype										
LBBAP	5	0.74 [0.55, 0.99]	0.04	0%	0.01 (including LVSP)/0.93 (excluding LVSP)	5	0.64 [0.41,1.02]	0.06	59%	0.64
LBBP	1	0.45 [0.09, 2.26]	0.33	/		2	0.89 [0.50, 1.60]	0.71	8%	
HBP	2	0.69 [0.16, 3.03]	0.62	10%		4	0.40 [0.12, 1.36]	0.14	53%	
LVSP	1	4.00 [1.63, 9.83]	0.003	/		/	/	/	/	
Mixed CSP Subtypes	7	0.80 [0.48, 1.33]	0.38	52%		6	0.74 [0.46, 1.19]	0.22	44%	
Patient Phenotype										
LVEF ≤ 35%&LBBB	4	0.94 [0.43, 2.05]	0.88	49%	0.84	5	0.58 [0.34, 0.97]	0.04	22%	0.65
Others	12	0.86 [0.56, 1.33]	0.49	51%		12	0.67 [0.47, 0.95]	0.02	55%	
Subgroup	Echocardiographic non-response					Echocardiographic super-response				
	Study	RR [95%CI]	*P* value	I^2^	*P* for interaction	Study	RR [95%CI]	*P* value	I^2^	*P* for interaction
Total	13	0.58 [0.41, 0.82]	0.002	70%		6	1.86 [1.43, 2.43]	< 0.00001	34%	
Study design										
RCT	2	0.58 [0.27, 1.24]	0.16	0%	0.98	1	12.0 [1.66, 86.77]	0.01	/	0.05
observational study	11	0.57 [0.39, 0.85]	0.005	75%		5	1.70 [1.45, 1.99]	< 0.00001	0%	
CSP subtype										
LBBAP	5	0.57 [0.30, 1.08]	0.09	82%	0.02 (including LVSP)/0.54 (excluding LVSP)	3	2.02 [1.17, 3.49]	0.01	56%	0.45
LBBP	3	0.64 [0.43, 0.96]	0.03	0%		1	1.56 [1.04, 2.35]	0.03	/	
HBP	2	0.24 [0.06, 0.96]	0.04	57%		1	2.84 [1.55, 5.21]	0.0007	/	
LVSP	1	1.21 [0.82, 1.80]	0.34	/		/	/	/	/	
Mixed CSP Subtypes	2	0.47 [0.25, 0.86]	0.02	0%		1	1.75 [0.58, 5.24]	0.32	/	
Patient Phenotype										
LVEF ≤ 35%&LBBB	7	0.46 [0.30, 0.69]	0.0002	40%	0.16	4	1.93 [1.32, 2.81]	0.0006	58%	0.91
Others	6	0.73 [0.43, 1.25]	0.25	76%		2	2.00 [1.17, 3.41]	0.01	0%	

#### 3.5.2 Sensitivity analysis

LOO analysis indicated limited robustness of the pooled estimate, as both the effect size and statistical significance varied with sequential exclusion of individual studies (Figure 3D).

#### 3.5.3 Publication bias

The funnel plot appeared symmetrical (Figure 4D), and Egger's test revealed insignificant publication bias (*P* = 0.500).

In absolute terms, CSP was associated with a 1.17% absolute reduction in ACM compared with BVP (10.65% vs. 11.82%), corresponding to a number needed to treat (NNT) of 86 to prevent one additional death. However, the wide CI around the relative effect estimate precludes a precise estimation of the NNT CI (Supplementary Table S4).

### 3.6 HFH

17 comparative groups comprising 3,979 patients (CSP: 2,121; BVP: 1,858) reported HFH (28, 33, 36, 39, 43, 45–48, 50, 52, 56, 58, 59, 61, 62). Pooled analysis of RRs demonstrated that CSP was associated with a significantly lower risk of HFH compared with BVP (RR: 0.65; 95% CI: 0.49–0.87; *P* = 0.004), with moderate heterogeneity (I^2^ = 50%, *P* = 0.004; Figure 2E).

Among the included studies, five reported HRs and nine reported RRs. Accordingly, subgroup analyses were conducted based on the type of effect measure. The pooled analysis of HRs demonstrated no statistically significant difference between CSP and BVP (HR: 0.73; 95% CI: 0.40–1.36; *P* = 0.32; I^2^ = 80%). Similarly, the pooled estimate based on RRs showed an insignificant difference (RR: 0.79; 95% CI: 0.53–1.19; *P* = 0.26; I^2^ = 25%). No statistically significant interaction was observed between subgroups stratified by effect measure type (*P* for interaction = 0.83; Figure 5B).

#### 3.6.1 Subgroup analyses

##### 3.6.1.1 Study design

No significant interaction was noted across RCTs and observational studies (*P* for interaction = 0.72). Five RCTs (397 patients) showed an insignificant difference (RR: 0.75; 95% CI: 0.35–1.59; *P* = 0.45; I^2^ = 3%). In contrast, twelve observational studies (3,582 patients) demonstrated a significant benefit favoring CSP (RR: 0.64; 95% CI: 0.47–0.88; *P* = 0.006; I^2^ = 60%).

##### 3.6.1.2 CSP subtype

Each CSP subtypes demonstrated non-significant trends. No significant difference was observed across CSP subtypes (*P* for interaction = 0.64).

##### 3.6.1.3 Patient phenotype

Significant reductions in HFH risk were observed both in patients meeting traditional CRT criteria (RR: 0.58; 95% CI: 0.34–0.97; *P* = 0.04; I^2^ = 22%) and in those with other indications (RR: 0.67; 95% CI: 0.47–0.95; *P* = 0.02; I^2^ = 55%), with insignificant interaction between subgroups (*P* for interaction = 0.65; Table 3).

#### 3.6.2 Sensitivity analysis

LOO analysis demonstrated that the pooled estimate remained stable, ranging from 0.46 to 0.93 after sequential exclusion of individual studies, indicating reasonable robustness (Figure 3E).

#### 3.6.3 Publication bias

The funnel plot appeared symmetrical (Figure 4E), and Egger's test showed no evidence of publication bias (*P* = 0.799).

On an absolute scale, the HFH rate was 11.46% in the CSP group versus 17.28% in the BVP group, corresponding to an absolute risk reduction of 5.88%. The associated number NNT was 17 (95% CI:13–28), indicating that one HFH event could be prevented for every 17 patients treated with CSP instead of BVP (Supplementary Table S4).

### 3.7 Echocardiographic Non-response

A total of 13 comparative groups comprising 2,087 patients (CSP: 910; BVP: 1,177) reported echocardiographic non-response (28, 30, 34, 38, 42, 44, 49–51, 56, 58). Pooled analysis demonstrated that CSP was associated with a significantly lower risk of non-response compared with BVP (RR: 0.58; 95% CI: 0.41–0.82; *P* = 0.002), with substantial heterogeneity (I^2^ = 70%, *P* < 0.0001; Figure 2F).

#### 3.7.1 Subgroup analyses

##### 3.7.1.1 Study design

No significant interaction was noted across RCTs and observational studies (*P* for interaction = 0.98). Two RCTs (102 patients) showed an insignificant difference (RR: 0.58; 95% CI: 0.27–1.24; *P* = 0.16; I^2^ = 0%). In contrast, eleven observational studies (1,985 patients) demonstrated a significant benefit favoring CSP (RR: 0.57; 95% CI: 0.39–0.85; *P* = 0.005; I^2^ = 75%).

##### 3.7.1.2 CSP subtype

LBBP (RR: 0.64; 95% CI: 0.43–0.96; *P* = 0.03; I^2^ = 0%), HBP (RR: 0.24; 95% CI: 0.06–0.96; *P* = 0.04; I^2^ = 57%), and mixed CSP (RR: 0.47; 95% CI: 0.25–0.86; *P* = 0.02; I^2^ = 0%) were each associated with significantly lower non-response rates. LBBAP showed a non-significant trend toward benefit (RR: 0.57; 95% CI: 0.30–1.08; *P* = 0.09; I^2^ = 82%). LVSP did not demonstrate a significant effect (RR: 1.21; 95% CI: 0.82–1.80; *P* = 0.34; single study). A significant difference across CSP subtypes was observed when LVSP was included (*P* for interaction = 0.02), which disappeared after excluding LVSP (*P* for interaction = 0.54), indicating that LVSP likely contributed substantially to heterogeneity.

##### 3.7.1.3 Patient phenotype

A significant benefit was observed among patients meeting traditional CRT criteria (RR: 0.46; 95% CI: 0.30–0.69; *P* = 0.0002; I^2^ = 40%). Patients with other indications demonstrated a non-significant trend toward benefit (RR: 0.73; 95% CI: 0.43–1.25; *P* = 0.25; I^2^ = 76%). No significant interaction was detected between subgroups (*P* for interaction = 0.16; Table 3).

#### 3.7.2 Sensitivity analysis

LOO analysis confirmed the robustness of the pooled estimate, as no individual study disproportionately influenced the overall effect (Figure 3F).

#### 3.7.3 Publication bias

The funnel plot suggested slight asymmetry (Figure 4F). Nevertheless, Egger's test did not indicate significant publication bias (*P* = 0.191).

Absolute rates of echocardiographic non-response were 17.69% in the CSP group versus 28.46% in the BVP group, corresponding to an absolute risk reduction of 10.77%. The number NNT was 9 (95% CI: 7–14), indicating that one case of non-response could be avoided for every 8 patients treated with CSP instead of BVP (Supplementary Table S4).

### 3.8 Echocardiographic super-response

6 comparative groups comprising 1,461 patients (CSP: 634; BVP: 827) reported echocardiographic super-response (28, 31, 42, 44, 50, 62). Pooled analysis demonstrated that CSP was associated with a significantly higher rate of super-response relative to BVP (RR: 1.86; 95% CI: 1.43–2.4; *P* < 0.00001), with low heterogeneity (I^2^ = 34%, *P* = 0.18; Figure 2G).

#### 3.8.1 Subgroup analyses

##### 3.8.1.1 CSP subtype

LBBAP (RR: 2.02; 95% CI: 1.17–3.49; *P* = 0.01; I^2^ = 56%), LBBP (RR: 1.56; 95% CI: 1.04–2.35; *P* = 0.03; single study), and HBP (RR: 2.84; 95% CI: 1.55–5.21; *P* = 0.0007; single study) were each associated with significantly higher super-response rates. Mixed CSP showed a non-significant trend toward benefit (RR: 1.75; 95% CI: 0.58–5.24; *P* = 0.32; single study). No significant interaction was observed across CSP subtypes (*P* for interaction = 0.45).

##### 3.8.1.2 Patient phenotype

Significant benefits were noted both in patients meeting traditional CRT criteria (RR: 1.93; 95% CI: 1.32–2.81; *P* = 0.0006; I^2^ = 58%) and in those with other indications (RR: 2.00; 95% CI: 1.17–3.41; *P* = 0.01; I^2^ = 0%), with insignificant interaction between subgroups (*P* for interaction = 0.91; Table 3).

#### 3.8.2 Sensitivity analysis

LOO analysis confirmed the robustness of the pooled estimate (Figure 3G).

#### 3.8.3 Publication bias

Publication bias was not assessed owing to the limited included studies (*n* < 10).

Absolute super-response rates were 40.22% with CSP versus 22.49% with BVP, corresponding to an absolute increase of 17.73% in favor of CSP. The number NNT to achieve one additional super-response was 6 (95% CI: 5–8), indicating that six patients would need to be treated with CSP instead of BVP to obtain one additional super-response (Supplementary Table S4).

### 3.9 Other related indicators

CSP was associated with significant improvements compared with BVP in LVEDV (MD: −19.09 mL; 95% CI: −28.73–−9.46 mL; *P* = 0.0001), LVESV (MD: −13.74 mL; 95% CI: −23.19–−4.28 mL; *P* = 0.004), LVEDD (MD: −2.90 mm; 95% CI: −4.26–−1.55 mm; *P* < 0.0001), LVESD (MD: −2.49 mm; 95% CI: −4.28–−0.70 mm; *P* = 0.003), and LAD (MD: −2.39 mm; 95% CI: −4.68–−0.11 mm; *P* = 0.04).

Differences were insignificant in pacing threshold (MD: −0.05 V; 95% CI: −0.19–0.09 V; *P* = 0.49), ventricular impedance (MD: −149.55 *Ω*; 95% CI: −371.82–72.72 Ω; *P* = 0.19), or R-wave amplitude (MD: 1.51 mV; 95% CI: −0.07–3.08 mV; *P* = 0.06).

CSP was associated with significantly shorter fluoroscopy time (MD: −5.04 min; 95% CI: −8.62–−1.45 min; *P* = 0.006), whereas total procedure time did not differ significantly (MD: −12.37 min; 95% CI: −28.05–3.31 min; *P* = 0.12).

No significant differences were observed in procedural complications, including pneumothorax (RR: 0.44; 95% CI: 0.13–1.53; *P* = 0.20), lead dislodgement/failure (RR: 0.61; 95% CI: 0.33–1.14; *P* = 0.12), pericardial effusion (RR: 0.55; 95% CI: 0.18–1.66; *P* = 0.29), or infection (RR: 0.71; 95% CI: 0.23–2.18; *P* = 0.55). These findings should be interpreted cautiously due to potential underreporting (Supplementary Table S5).

### 3.10 GRADE assessment

The certainty of evidence ranged from very low to low (Supplementary Table S6). The primary outcome, change in LVEF (34 comparative groups), was rated as very low certainty due to substantial heterogeneity (I^2^ = 72%). Although the pooled estimate demonstrated significant improvement (MD: 4.22%; 95% CI: 2.74%–5.7%), serious inconsistency reduced confidence in the estimate. Similarly, evidence for QRS duration (30 studies; I^2^ = 83%) and HFH (17 studies; I^2^ = 50%) was rated as very low due to serious inconsistency. HFH (RR: 0.65; 95% CI: 0.49–0.87) was further downgraded for imprecision. ACM was also rated as very low certainty. Although inconsistency was not serious, the CI (RR: 0.87; 95% CI: 0.62–1.22) crossed the null and encompassed both potential benefit and harm, warranting downgrading for imprecision. Two outcomes were rated as low certainty. Change in NYHA functional class (14 studies; MD: −0.34; 95% CI: −0.47–−0.21) showed no serious limitations across domains, resulting in a low rating based on the observational design. Echocardiographic super-response (6 studies; RR: 1.86; 95% CI: 1.43–2.43) was also rated as low certainty, given consistent findings without serious inconsistency or imprecision. No outcomes were upgraded. In all cases, plausible confounding was unlikely to reduce the observed effects, and there was no evidence of a sufficiently large magnitude of effect or dose-response gradient to warrant upgrading the certainty of evidence.

## 4 Discussion

CRT using BVP has proven effective in the population with left ventricular dysfunction, notably in individuals with LBBB (63). However, clinical experience has demonstrated a relatively high nonresponse rate to BVP-based CRT (BVP-CRT) and limited benefit in patients with a normal QRS duration or concomitant right bundle branch block (RBBB) (64, 65). LBBAP restores electrical synchrony and improves cardiac structure and function in LBBB patients (66, 67). Compared with HBP, LBBAP exhibits superior pacing parameters and comparable clinical outcomes, while outperforming conventional BVP (38, 61). CSP has thus attracted increasing attention and undergone rapid advancement due to its more favorable electrophysiological profile and simplified implantation technique, effectively addressing the technical limitations associated with HBP. Therefore, this systematic review and meta-analysis evaluated the comparative clinical efficacy of CSP versus BVP in CRT among HF patients.

### 4.1 Summary of main findings

This meta-analysis systematically compared the efficacy and safety of CSP versus BVP in CRT, incorporating 35 studies and 7,019 patients. The principal findings can be summarized as follows: (1) Echocardiographic remodeling: CSP was associated with significant improvements in LVEF (MD: 4.22%; 95% CI: 2.74%–5.70%), LVEDV, LVESV, LVEDD, LVESD, and LAD compared with BVP. (2) Electrical and functional outcomes: CSP significantly shortened QRS duration (MD: −19.6 ms; 95% CI: −24.18–−15.02 ms), improved NYHA functional class (MD: −0.34; 95% CI: −0.47–−0.21), and lowered HFH risk (RR: 0.65; 95% CI: 0.49–0.87). (3) Response rates: CSP significantly increased the rate of echocardiographic super-response (RR: 1.86; 95% CI: 1.43–2.43) and reduced the risk of non-response (RR: 0.58; 95% CI: 0.41–0.82). (4) ACM: No significant difference in ACM was observed between groups. (5) Procedural safety: Rates of pneumothorax, lead dislodgement, pericardial effusion, and infection were comparable. CSP was associated with shorter fluoroscopy time (MD: −5.04 min; 95% CI: −8.62–−1.45 min). (6) Overall, these findings provide comprehensive evidence supporting the use of CSP in CRT. However, interpretation should be tempered by the overall low certainty of evidence according to GRADE (Section 3.10).

### 4.2 Interpretation of primary outcomes

Improvement in LVEF remains central to assessing CRT efficacy. CSP was associated with an absolute increase in LVEF of 4.22% relative to BVP (MD: 4.22%; 95% CI: 2.74%–5.70%), representing a clinically meaningful magnitude of reverse remodeling. Prior evidence from the RELAX-AHF-2 trial suggests that each 5% increment in LVEF corresponds to an approximate 5% reduction in the composite risk of cardiovascular death or HF/renal rehospitalization at 180 days (68). Substantial heterogeneity was observed (I^2^ = 72%). Study design emerged as a key contributor: ten RCTs demonstrated a non-significant trend favoring CSP (MD: 1.46%; *P* = 0.22), whereas 24 observational studies showed a significant benefit (MD: 5.37%; *P* < 0.00001). The significant interaction (*P* for interaction: 0.008) suggests that treatment effects differ by study design. Observational estimates may be influenced by selection bias, residual confounding, and operator experience. Accordingly, conclusions regarding LVEF should primarily rely on randomized evidence, with caution that observational data may overestimate the effect size.

Shortening of QRS duration reflects improved ventricular electrical synchrony. Our analysis demonstrated that CSP reduced QRS duration by 19.60 ms, an effect consistently observed across LBBAP (MD: −23.53 ms), LBBP (MD: −25.22 ms), and HBP (MD: −21.95 ms) subtypes, whereas LVSP showed insignificant benefit (MD: −10.32 ms; *P* = 0.33). Importantly, LVSP, representing septal pacing without confirmed conduction system capture, achieved inferior electrical resynchronization compared with definitive CSP. This underscores a critical procedural principle: when performing LBBAP, operators should aim for confirmed LBBP rather than non-selective septal pacing (LVSP).

HFH is a pivotal clinical endpoint in CRT. Subgroup analyses stratified by effect measure demonstrated that the apparent benefit of CSP on HFH did not reach statistical significance in studies reporting HRs (HR: 0.73; 95% CI 0.40–1.36; *P* = 0.32; I^2^ = 80%) or in those reporting RRs (RR: 0.79; 95% CI 0.53–1.19; *P* = 0.26; I^2^ = 25%), with insignificant interaction between subgroups (*P* for interaction=0.83). These findings indicate that the overall pooled reduction in HFH risk (RR: 0.65; 95% CI 0.49–0.87; *P* = 0.004) should be interpreted cautiously, as it may be influenced by substantial heterogeneity among HR-based studies and the methodological implications of combining different effect measures. Further subgroup analyses suggested more pronounced and consistent benefits among patients meeting traditional CRT criteria (LVEF ≤ 35% with LBBB; RR: 0.58; 95% CI 0.34–0.97; I^2^ = 22%), suggesting that CSP confers clearer and more reproducible benefits in this well-defined population. This observation aligns with prior evidence and underscores the importance of stringent patient selection in optimizing therapeutic outcomes. Nevertheless, prespecified subgroup analyses did not fully account for the observed heterogeneity. Although sensitivity analyses supported a degree of robustness, the presence of substantial variability warrants cautious interpretation. Future rigorously designed RCTs involving more homogeneous patient populations are needed to definitively establish the effect of CSP on HFH reduction.

To facilitate clinical interpretation of the relative benefits observed in this meta-analysis, we calculated absolute event rates and corresponding numbers NNT for key outcomes. The NNT was 6 for echocardiographic super-response, 9 for non-response, 17 for HFH, and 86 for ACM. These estimates follow a clinically intuitive gradient: the most readily achievable benefit is improvement in echocardiographic markers of reverse remodeling (NNT: 6), followed by reduction in hard clinical events such as HFH (NNT: 17), whereas the effect on mortality, although directionally favorable, requires treatment of a substantially larger number of patients (NNT: 86). In context, these NNT values compare favorably with those reported for other established heart failure therapies. For instance, the NNT for sacubitril/valsartan versus enalapril to prevent one cardiovascular death or HFH in the PARADIGM-HF trial was approximately 21 over 27 months, and the NNT for guideline-directed medical therapy to prevent one death in chronic HFrEF generally ranges from 20 to 50. Against this background, the NNT of 17 for HFH prevention with CSP appears clinically meaningful, particularly as an incremental benefit on top of standard medical therapy. Nevertheless, these estimates should be interpreted with caution, given the predominantly observational nature of the included studies, the presence of substantial heterogeneity, and potential publication bias underlying the pooled relative effects. Accordingly, they should be regarded as hypothesis-generating and warrant confirmation in adequately powered RCTs.

### 4.3 Clinical implications of subgroup analyses

Comparison of CSP subtypes constituted a central component of the subgroup analyses and yielded clinically meaningful insights. For LVEF improvement, LBBAP (MD: 6.28%), LBBP (MD: 6.48%), and HBP (MD: 5.13%) were each significantly superior to BVP, whereas LVSP demonstrated no benefit (MD: −1.90%). Similarly, for QRS shortening, LBBAP, LBBP, and HBP were consistently superior to BVP, while LVSP was ineffective. With respect to ACM, LBBAP was associated with reduced mortality (RR: 0.74), mixed CSP showed a favorable trend (RR: 0.80), and LVSP was associated with significantly increased mortality (RR: 4.00). A significant interaction across CSP subtypes was observed when LVSP was included (*P* for interaction: 0.01), which disappeared after its exclusion (*P* for interaction: 0.93), indicating that LVSP was a major contributor to heterogeneity. The foregoing findings convey a clear clinical message: definitive conduction system capture (LBBP or HBP) appears essential for achieving meaningful clinical benefit. LVSP, although technically categorized under LBBAP, lacks confirmed conduction system engagement and should not be regarded as equivalent to true CSP modalities.

Subgroup analyses by patient phenotype further demonstrated that patients meeting traditional CRT criteria (LVEF ≤ 35% with LBBB) exhibited numerically greater improvements in LVEF (MD: 4.76% vs. 3.88%), HFH reduction (RR: 0.58 vs. 0.67), and decreased non-response (RR: 0.46 vs. 0.73), although interaction tests were not statistically significant (all *P* for interaction > 0.16). This pattern is consistent with the established CRT literature, in which LBBB with prolonged QRS duration is the strongest predictor of response. Importantly, patients with non-traditional indications, including pacing-induced cardiomyopathy, LVEF 35%–50%, and non-LBBB conduction disturbances, also appeared to derive benefit from CSP. This observation suggests that CSP may extend physiologic resynchronization to populations that respond suboptimally to conventional BVP.

Subgroup analysis by study design showed significant interactions for improvements in LVEF (*P* = 0.008), NYHA class (*P* = 0.008), and QRS duration (*P* = 0.001), all of which were better in observational studies. However, for hard endpoints (i.e., HFH, ACM, non-response), treatment effects were directionally consistent between RCTs and observational studies, with non-significant interactions (*P* > 0.70). These findings supported the robustness of the estimates of clinical outcomes, while the limited number of related RCTs may be a key limitation. Future large-scale randomized trials are needed to definitively establish the comparative effectiveness of CSP.

### 4.4 Clinical implications of secondary outcomes and other indicators

Echocardiographic super-response and non-response represent integrated measures of CRT efficacy. CSP was associated with a 86% increase in super-response (RR: 1.86) and a 42% reduction in non-response (RR: 0.58), effects that were broadly consistent across CSP subtypes. Given that super-response correlates with favorable long-term prognosis, whereas non-response predicts adverse outcomes, these findings further reinforce the clinical value of CSP.

Regarding procedural characteristics, CSP was associated with shorter fluoroscopy time (MD: −5.04 min), likely reflecting avoidance of coronary sinus venography and branch vessel selection. Total procedure time did not differ significantly (MD: −12.37 min; P: 0.12), possibly due to the learning curve associated with CSP and variability in operator experience. No significant differences were observed in pacing threshold, ventricular impedance, or R-wave amplitude, indicating comparable short-term electrical performance between CSP and BVP.

With respect to complications, CSP demonstrated numerically lower risks of pneumothorax, lead dislodgement, pericardial effusion, and infection, although differences were not statistically significant. Notably, the point estimate for infection risk (RR: 0.71), if confirmed in larger cohorts, would be clinically meaningful, given the substantial morbidity and healthcare burden associated with device-related infection. Mechanistically, reduced subclavian vein punctures and avoidance of coronary sinus manipulation may contribute to this potential advantage.

### 4.5 Comparison with previous meta-analyses

Our findings largely align with those of Gin et al. (18) in several key aspects. First, with respect to electrical synchrony, CSP was associated with significant QRS duration shortening in the present analysis (MD: −19.60 ms; 95% CI: −24.18–−15.02 ms), closely paralleling the reduction observed by Gin et al. for CSP versus BVP (MD: −20.3 ms; 95% CI: −26.1–−14.5 ms). This improvement likely reflects direct engagement of the His-Purkinje conduction system, thereby achieving ventricular activation that more closely approximates physiological resynchronization compared with the epicardial activation pathway utilized in BVP. Second, regarding cardiac functional recovery, our pooled analysis demonstrated that CSP significantly improved LVEF (MD 4.22%; 95% CI: 2.74% to 5.70%) and reduced NYHA functional class (MD: −0.34; 95% CI: −0.47–−0.21). These findings are highly comparable to those of Gin et al., who reported improvements in LVEF (MD 5.2%; 95% CI: 3.5% to 6.9%) and NYHA class (MD: −0.40; 95% CI: −0.6–−0.2) with CSP. Third, in terms of procedural safety, our study identified insignificant differences between CSP and BVP in the incidence of pneumothorax, lead dislodgement or failure, pericardial effusion, or infection. This is consistent with Gin et al., who similarly reported insignificant difference in overall perioperative complications [CSP: 29/539 [5.4%] vs. BVP: 38/572 [6.6%]; *P* = 0.44], with lead dislodgement representing the most frequent adverse event.

However, our findings diverged from those of Gin et al. with respect to pacing parameters. In our pooled analysis, no statistically significant differences were observed between CSP and BVP in pacing threshold, ventricular impedance, or R-wave amplitude. By contrast, Gin et al. reported that LBBAP was associated with a significantly lower pacing threshold (MD: −0.51 V; 95% CI: −0.65–−0.37 V; *P* < 0.05), whereas HBP demonstrated a trend toward a higher pacing threshold (MD: 0.62 V; 95% CI: −0.03–1.26 V; *P* > 0.05). These inconsistencies may be attributable to statistical heterogeneity arising from the inclusion of newly published studies, the procedural learning curve inherent to emerging CSP techniques, and differences in follow-up duration.

In comparison to Gin et al.'s meta-analysis, our study identified several novel differences between CSP and BVP: First, the observed reduction in the risk of HFH provides additional evidence supporting CSP as a viable alternative to BVP. Second, CSP was associated with a significantly shorter fluoroscopy time, although total procedural duration did not differ significantly between groups. Third, CSP conferred more pronounced reverse cardiac remodeling, as evidenced by significant reductions in LVEDV, LVESV, LVEDD, and LVESD. Finally, CSP was related to a significantly higher rate of echocardiographic super-response (RR: 1.86) and a lower rate of echocardiographic non-response (RR: 0.58).

### 4.6 Mechanistic discussion

The differential clinical outcomes observed among CSP subtypes, particularly the absence of significant benefit with LVSP, may be attributable to the distinct underlying electrophysiological mechanisms elaborated below.

BVP-CRT involves the placement of pacing leads at the right ventricular apex and within a coronary sinus branch (typically targeting the lateral or posterior wall of the left ventricle). The system senses atrial activity and delivers pacing stimuli to the right and left ventricles either simultaneously or in an optimized sequence to achieve ventricular resynchronization. Although clinically effective, this excitation sequence remains partly non-physiological. By contrast, CSP, via direct capture of the HBB or LBB, restores or closely mimics physiological ventricular activation. This approach markedly improves biventricular electromechanical synchrony, enhances cardiac pump efficiency (as evidenced by increased LVEF and cardiac output), mitigates functional mitral regurgitation, prevents or reverses maladaptive remodeling, and may indirectly modulate pathological neuroendocrine activation.

Qian et al. (69) conducted high-density left ventricular pacing studies in a porcine model to evaluate the effects of different pacing sites. They observed that the difference in total left ventricular activation time compared with intrinsic sinus rhythm was not statistically significant with LBBP, significantly favoring LBBP over conventional right ventricular pacing. Chen et al. (70) analyzed 20 individuals with complete AV block who underwent acute-phase pacing using right ventricular apical pacing (RVAP), right ventricular septal pacing (RVSP), HBP, and LBBP. They compared electrocardiographic parameters and echocardiographic indices of synchrony, demonstrating that both HBP and LBBP yielded superior electro-mechanical synchronization compared to RVAP. Furthermore, they proposed that the time to peak of the septal-to-lateral wall activation time (Sti-LVAT) possibly be a surrogate marker for left ventricular systolic function and mechanical synchrony. Consistent clinical findings indicate that patients with LBBB derive the greatest benefit and exhibit the highest response rate to CRT compared with those with other intraventricular conduction disturbances (e.g., RBBB or nonspecific intraventricular delays). This superior responsiveness arises from the characteristic pattern of dyssynchrony in LBBB, namely, a pronounced delay in left ventricular lateral wall activation, which is optimally corrected through targeted left ventricular pacing.

The limited benefit observed in the LVSP subgroup primarily arises from its non-physiological activation pattern. LVSP directly stimulates the left ventricular septum without engaging the intrinsic conduction system. Therefore, depolarization originates at the septal pacing site and propagates slowly through the adjacent myocardium before extending to the apical and free walls. The left ventricular free wall, particularly the basal region, is activated last, failing to address the fundamental electromechanical dyssynchrony underlying HF. This limitation is especially evident in patients with HF and concomitant LBBB, in whom LVSP induces premature septal activation while right ventricular activation remains reliant on the native AV node-His-RBB pathway. The result is an aberrant activation sequence, characterized by early left septal contraction preceding right ventricular contraction, opposite to the physiological sequence and distinct from the pathological activation order of LBBB. This iatrogenic interventricular dyssynchrony may counteract or even negate the therapeutic benefit of resynchronization therapy, further impairing cardiac function (71, 72).

Although CSP has emerged as a promising alternative to conventional BVP, the mechanisms underlying its therapeutic efficacy remain incompletely elucidated. Chen et al. (31) reported that the remodeling response to LBBAP and BVP in nonischemic cardiomyopathy was modulated by septal scar burden; extensive septal scarring was associated with worse prognosis and unresponsiveness to CRT. Preoperative assessment of myocardial substrate using cardiac magnetic resonance may thus be crucial for optimizing resynchronization strategies and long-term risk stratification. Richter et al. (73) applied three-dimensional electroanatomical mapping (EAM) to guide LBBAP implantation in patients with structural heart disease and advanced conduction abnormalities, achieving near-zero fluoroscopy exposure and high procedural success rates. No intraoperative complications requiring intervention were observed. Postoperatively, QRS duration was significantly shortened, and capture thresholds remained low. During mid-term follow-up, pacing thresholds were stable, accompanied by marked improvements in LVEF and NYHA class. These findings highlight the value of electrophysiological mapping in identifying the latest activated ventricular region and optimizing the LBBAP target site to maximize therapeutic benefit. Future research should aim to identify predictors of CSP nonresponse and hyperresponse, refine individualized patient selection criteria, and integrate imaging markers (e.g., myocardial scar burden) with electrophysiological parameters to optimize patient stratification and improve long-term outcomes.

### 4.7 Limitations

The findings of this meta-analysis should be interpreted with caution, given the quality of the underlying evidence base. According to the GRADE framework, the overall certainty of evidence ranged from very low to low. The primary limitation lies in the predominance of observational studies (25 of 35), which are inherently susceptible to selection bias, residual confounding, and measurement bias. Importantly, only ten RCTs were included in this meta-analysis, the majority of which were small and individually underpowered. For key outcomes, such as ACM and HFH, subgroup analyses restricted to RCTs yielded non-significant results with wide CIs, underscoring the lack of robust randomized evidence to support definitive conclusions. Although several outcomes demonstrated consistent directional effects, substantial statistical heterogeneity, reflected by I^2^ values exceeding 70% for key endpoints such as LVEF and QRS duration, indicates meaningful variability across studies in patient characteristics, procedural techniques, and outcome assessment methods. Such inconsistency diminishes the robustness of pooled estimates. Furthermore, imprecision in effect estimates, particularly for critical outcomes such as ACM, further tempers our confidence. The wide CIs surrounding the mortality estimate, encompassing both the possibility of no effect and a clinically meaningful benefit, preclude definitive conclusions regarding its impact on survival. Importantly, no outcomes were upgraded under GRADE criteria, as large effect sizes or dose-response relationships were absent. Consequently, although CSP appears associated with improvements in surrogate markers such as LVEF and NYHA class, the true magnitude of benefit, especially for patient-important outcomes including ACM and HFH, remains uncertain.

Besides the limitations identified by the GRADE assessment, several methodological and clinical limitations merit consideration.

First, despite performing stratified analyses by study design and interaction testing, biases intrinsic to observational research cannot be fully eliminated. Therefore, estimates for hard clinical endpoints, particularly ACM, should be regarded as hypothesis-generating and require confirmation in adequately powered RCTs. Although the DerSimonian and Laird random-effects model was used for consistency, it may yield overly narrow CIs in the presence of substantial heterogeneity compared with more robust estimators such as restricted maximum likelihood. Publication bias was detected for LVEF (Egger's test *P* < 0.05); although trim-and-fill adjustment continued to favor CSP, the true effect size may be smaller than originally estimated. For outcomes with fewer than 10 studies (e.g., super-response), formal publication bias assessment was not feasible, and findings should be interpreted cautiously.

Second, considerable clinical heterogeneity existed across studies with respect to CSP definitions, procedural protocols, operator experience, follow-up duration, and outcome measurement. Subgroup classification relied on original study reports and may involve some degree of overlap or misclassification. Certain subgroups, including RCTs and LVSP, contained a limited number of studies, restricting statistical power for interaction testing and potentially compromising subgroup stability. Moreover, operator learning-curve effects were insufficiently addressed. CSP techniques, particularly LBBP, have substantial learning curves, and early-phase procedural experience may have led to underestimation of efficacy and overestimation of complication rates. The absence of patient-level data further limited exploration of individual predictors of response; variables such as age, sex, ischemic etiology, myocardial scar burden, atrial fibrillation burden, and ventricular pacing percentage could not be adequately adjusted for at the aggregate meta-analytic level.

Third, reporting of safety outcomes was incomplete and inconsistent. Only a minority of studies provided a detailed characterization of adverse events, raising concerns regarding potential underreporting of CSP-related complications. Additionally, several studies did not clearly describe loss to follow-up, introducing possible attrition bias that may overestimate treatment effects and reduce internal validity, thereby limiting generalizability.

Finally, long-term evidence remains sparse. Few studies reported follow-up beyond 36 months, resulting in limited data regarding sustained efficacy and long-term safety, including lead durability and late adverse events. Critical endpoints such as ACM, HFH, device-related complications, and lead performance stability require follow-up of at least five years for comprehensive evaluation. Robust long-term data are essential to inform clinical decision-making and support broader adoption of CSP. Future prospective investigations should therefore incorporate sufficiently extended follow-up to address this important evidence gap.

In summary, high-quality prospective cohort studies and, ideally, large-scale, multicenter RCTs with standardized protocols and long-term follow-up are urgently needed to provide definitive evidence. These findings underscore the gap between promising surrogate improvements and the level of evidence required to guide clinical practice.

## 5 Conclusion

Compared with BVP, CSP appears to be associated with greater improvements in echocardiographic and electrocardiographic parameters, as well as reductions in HFH, suggesting its potential as a more effective CRT modality. Therefore, CSP may be a promising alternative therapeutic strategy for patients with HF. However, given the substantial heterogeneity, potential publication bias, and predominance of non-randomized data, these conclusions should be interpreted cautiously. Importantly, the current evidence base—dominated by observational studies and limited by substantial heterogeneity and imprecision—remains insufficient to mandate a paradigm shift in clinical practice. The promising findings for CSP should be considered hypothesis-generating, and their adoption into routine clinical practice awaits confirmation from large, well-designed RCTs.

## Data Availability

The original contributions presented in the study are included in the article/Supplementary Material, further inquiries can be directed to the corresponding author.

## Supplementary material

Supplementary Figure S1 Detailed search strategy.

Supplementary Figure S2 Cochrane Handbook for Systematic Reviews of Interventions 5.1.0 was used to assess the RCTs' quality.

Supplementary Figure S3 Forest plots of outcomes after excluding studies with converted data (random-effects model). (A) change in LVEF and (B) change in QRS duration.

Supplementary Figure S4 Trim-and-fill funnel plot: change in LVEF.

Supplementary Table S1 Endpoint definitions.

Supplementary Table S2 The cohort study used the Newcastle-Ottawa Scale (NOS) for quality assessment.

Supplementary Table S3 Studies requiring data conversion: LVEF (4 studies) and QRS duration (5 studies). Data are presented as mean ± SD (white cells), median (range) (yellow cells), or mean (95% CI) (green cells).

Supplementary Table S4 Absolute event rates and NNT for key clinical outcomes.

Supplementary Table S5 Comparison of CSP with BVP in other clinical outcomes.

Supplementary Table S6 GRADE rating of each outcome.
