# Supplementary material for: Conduction system pacing compared with biventricular pacing for cardiac resynchronization therapy: a systematic review and meta-analysis
Source: Front Cardiovasc Med. 2026 Jun 5;13:1776637. doi: 10.3389/fcvm.2026.1776637 (PMC13278982; doi:10.3389/fcvm.2026.1776637)
Supplement: Supplementary Table S6 — GRADE rating of each outcome. [file Datasheet1.docx]

**Table S1** **Endpoint definitions**.

| Endpoint | Definition |
| --- | --- |
| ACM | All-cause mortality (ACM) was defined as death from any cause during the follow-up period, without exception across studies. |
| HFH | Heart failure hospitalization (HFH) was consistently defined as an unplanned hospital admission lasting >24 hours primarily for the management of worsening heart failure, requiring intravenous diuretic therapy or other intensive heart failure treatment. |
| Echocardiographic non-response | Echocardiographic non-response was uniformly defined as an absolute increase in left ventricular ejection fraction (LVEF) of <5% from baseline to follow-up across all 13 comparative groups reporting this outcome. |
| Echocardiographic super-response | Echocardiographic super-response was defined either as an absolute increase in LVEF ≥20% from baseline or as recovery of LVEF to ≥50% at follow-up. |

|  | **Echocardiographic Non-response**  **(13 comparative groups)** | **Echocardiographic Super-response**  **(6 comparative groups)** |
| --- | --- | --- |
| Chen 2022 | ΔLVEF<5% | ΔLVEF≥20% or LVEF ≥50% |
| Chen 2024a | ΔLVEF<5% | ΔLVEF≥20% or LVEF ≥50% |
| Moriña‐Vázquez 2022 | ΔLVEF<5% | ΔLVEF≥20% |
| Pujol-López 2022 | ΔLVEF<5% | ΔLVEF≥20% or LVEF ≥50% |
| Chen 2024b | ΔLVEF<5% | / |
| Guo 2020 | ΔLVEF<5% | / |
| Li 2020 | ΔLVEF<5% | / |
| Upadhyay 2019 | ΔLVEF<5% | / |
| Vijayaraman 2023-1 | ΔLVEF<5% | ΔLVEF≥20% or LVEF ≥50% |
| Vijayaraman 2023-2 | ΔLVEF<5% | / |
| Wang 2024 | ΔLVEF<5% | / |
| Zhu 2024a | ΔLVEF<5% | / |
| Zhu 2024b | ΔLVEF<5% | / |
| Zizek 2025 | / | LVEF ≥50% |

**Table S2** The cohort study used the Newcastle-Ottawa Scale (NOS) for quality assessment.

| **Study(25)** | **Selection** | | | | **Comparability** | | | **Outcome** | | | **Quality**  **Score** |
| --- | --- | --- | --- | --- | --- | --- | --- | --- | --- | --- | --- |
|  | **Representativeness** | **Selection of**  **non-exposed** | **Ascertainment**  **of exposure** | **Outcome not present at start** | **Comparability on most important factors** | **Comparability on other risk factors** | **Assessment of outcome** | | **Long enough follow-up (median≥6 months)** | **Adequacy**  **(completeness) of follow-up** |  |
| Chen et al. 2024a | * | * | * | * | * | - | * | | * | * | 8 |
| Ma et al. 2021 | * | * | * | * | * | - | - | | * | * | 7 |
| Moriña‐Vázquez et al. 2022 | - | - | * | * | * | - | - | | * | * | 5 |
| Chen et al. 2022 | * | * | * | * | * | - | * | | * | * | 8 |
| Tang et al. 2024 | * | * | * | * | * | - | - | | * | * | 7 |
| Vijayaraman et al. 2023-1 | * | * | * | * | * | - | - | | * | * | 7 |
| Zhu et al. 2024 | * | * | * | * | * | - | * | | * | * | 8 |
| Vijayaraman et al. 2024 | * | * | * | * | - | - | - | | * | - | 5 |
| Ma et al. 2024 | * | * | * | * | * | - | - | | * | * | 7 |
| Pujol-López et al. 2022 | - | - | * | * | * | - | - | | * | * | 5 |
| Tan et al. 2023 | * | * | * | * | * | - | - | | * | - | 6 |
| Wang et al. 2024 | * | * | * | * | * | - | * | | * | * | 8 |
| Kato et al. 2022 | * | * | * | * | * | - | * | | * | * | 8 |
| Shroff et al. 2024 | * | * | * | * | * | - | * | | * | * | 8 |
| Chen et al. 2023 | * | * | * | * | * | - | * | | * | * | 8 |
| Senes et al. 2021 | * | * | * | * | * | - | - | | * | * | 7 |
| Li et al. 2020 | * | - | * | * | * | - | * | | * | * | 7 |
| Liang et al. 2024 | * | * | * | * | * | - | - | | * | * | 7 |
| Wu et al. 2021 | * | * | * | * | * | - | * | | * | * | 8 |
| Diaz et al. 2024 | * | * | * | * | * | - | - | | * | * | 7 |
| Ezzeddine et al. 2023 | - | - | * | * | * | - | - | | * | * | 5 |
| Chen et al. 2024b | * | * | * | * | * | - | * | | * | - | 7 |
| Guo et al. 2020 | * | * | * | * | * | - | * | | * | - | 7 |
| Wang et al. 2020 | * | * | * | * | * | - | - | | * | * | 7 |
| Herweg et al. 2024 | * | * | * | * | - | - | - | | * | * | 6 |
|  | | | | | | | | | | | |

**Table S3** Studies requiring data conversion: LVEF (4 studies) and QRS duration (5 studies). Data are presented as mean±SD (white cells), median (range) (yellow cells), or mean (95% CI) (green cells).

| Author | Initial LVEF % | | Final LVEF % | | ∆ LVEF % | |
| --- | --- | --- | --- | --- | --- | --- |
|  | on | off | on | off | on | off |
| Li 2020 | 29.3±5.9 |  | 44.3±8.7 | 35.0±10.5 | 17.10 (10.8-20.4) | 7.0 (1.0-11.0) |
| Moriña‐Vázquez 2022 | 30 (28-34) | 30 (29-35) | 55 (45-60) | 40 (35-48) |  |  |
| Upadhyay 2019 | 28.0 (23.0-34.0) | 27.7 (23.6-30.7) | 34.6(30.8-45.0) | 32 (30.9-40.1) |  |  |
| Zizek 2025 |  |  |  |  | 14 (95%CI:11.2-16.8) | 8.5 (95%CI:5.6-11.2) |
|  |  |  |  |  |  |  |
|  |  |  |  |  |  |  |
|  |  |  |  |  |  |  |
| Author | Initial QRS duration(ms) | | Final QRS duration(ms) | | ∆QRS duration(ms) | |
|  | on | off | on | off | on | off |
| Diaz 2024a | 160 (150-184) | 160 (144-176) | 117.5±16.6 | 150.3±27 |  |  |
| Diaz 2024b | 165 (130.5-199.3) | 160 (144-176) | 144.9±15.8 | 150.3±27 |  |  |
| Li 2020 | 177.9±18.8 |  | 121.8±10.8 | 158.2±21.5 | -58 (-65.3- 40.0) | -12.5 (-40.0- 0) |
| Moriña‐Vázquez 2022 | 160 (150-160) | 160 (150-160) | 135 (120-145) | 140 (130-150) |  |  |
| Zizek 2025 |  |  |  |  | -33 (95%CI:-40,-26) | -32 (95%CI:-39,-25) |

**Table S4.** Absolute event rates and NNT for key clinical outcomes.

| **Outcome** | **CSP event rate** | **BVP event rate** | **Absolute risk difference** | **NNT (point estimate)** | **95%CI** |
| --- | --- | --- | --- | --- | --- |
| All-cause mortality | 10.65%(221/2076) | 11.82%(214/1811) | 1.17% | 86 | 32 to ∞ |
| Heart failure hospitalization | 11.40%(243/2121) | 17.28%(321/1858) | 5.88% | 17 | 13-28 |
| Echocardiographic non-response | 17.69%(161/910) | 28.46%(335/1177) | 10.77% | 9 | 7-14 |
| Echocardiographic super-response | 40.22%(255/634) | 22.49%(186/827) | 17.73% | 6 | 5-8 |

**Table S5** Comparison of CSP with BVP in other clinical outcomes.

| Outcomes | comparative | Participants | CSP | BVP | Statistical Method | Effect Estimate | P |
| --- | --- | --- | --- | --- | --- | --- | --- |
|  | groups |  |  |  |  |  |  |
| Echocardiographic outcomes |  |  |  |  |  |  |  |
| change in LVEDV, ml | 9 | 735 | 331 | 404 | MD(IV,Random,95%CI) | -19.09 [-28.73, -9.46] | 0.0001 |
| change in LVESV, ml | 10 | 736 | 327 | 409 | MD(IV,Random,95%CI) | -13.74 [-23.19, -4.28] | 0.004 |
| change in LVEDD, mm | 16 | 2358 | 1387 | 971 | MD(IV,Random,95%CI) | -2.90 [-4.26, -1.55] | <0.0001 |
| change in LVESD, mm | 8 | 819 | 406 | 413 | MD(IV,Random,95%CI) | -2.49 [-4.28, -0.70] | 0.003 |
| change in LAD, mm | 4 | 332 | 187 | 145 | MD(IV,Random,95%CI) | -2.39 [-4.68, -0.11] | 0.04 |
| Procedural outcomes |  |  |  |  |  |  |  |
| change in threshold LV/His lead, V | 10 | 1660 | 1162 | 498 | MD(IV,Random,95%CI) | -0.05 [-0.19, 0.09] | 0.49 |
| change in ventricular impedance,Ω | 4 | 213 | 100 | 113 | MD(IV,Random,95%CI) | -149.55 [-371.82, 72.72] | 0.19 |
| change in amplitude of R wave, mV | 4 | 281 | 134 | 147 | MD(IV,Random,95%CI) | 1.51 [-0.07, 3.08] | 0.06 |
| Fluoroscopy time, min | 12 | 3313 | 1704 | 1609 | MD(IV,Random,95%CI) | -5.04 [-8.62, -1.45] | 0.006 |
| Operation time, min | 11 | 2265 | 869 | 1396 | MD(IV,Random,95%CI) | -12.37 [-28.05, 3.31] | 0.12 |
| Procedural complications |  |  |  |  |  |  |  |
| pneumothorax | 4 | 3531 | 2000 | 1531 | RR(M-H,Random,95%CI) | 0.44 [0.13, 1.53] | 0.2 |
| lead dislodgment/failure | 9 | 4017 | 2243 | 1774 | RR(M-H,Random,95%CI) | 0.61 [0.33, 1.14] | 0.12 |
| pericardial effusion | 3 | 3491 | 1980 | 1511 | RR(M-H,Random,95%CI) | 0.55 [0.18, 1.66] | 0.29 |
| infection | 5 | 3623 | 2046 | 1577 | RR(M-H,Random,95%CI) | 0.71 [0.23, 2.18] | 0.55 |
|  |  |  |  |  |  |  |  |

**Table S6** GRADE rating of each outcome.

| Outcomes | No. of studies | Estimate [95%CI] | I^2^, P value | Risk of bias | Inconsistency | Indirectness | Imprecision | Publication bias | Plausible confounding | Magnitude of effect | Dose-response gradient | GRADE |
| --- | --- | --- | --- | --- | --- | --- | --- | --- | --- | --- | --- | --- |
| Change in LVEF | 34 | MD 4.22% [2.74, 5.70] | 72%; P <0.00001 | No serious risk | Serious inconsistency | No serious  indirectness | No serious imprecision | Strongly suspected | Would not reduce effect | No | No | Very low |
| Change in NYHA functional class | 14 | MD -0.34 [-0.47, -0.21] | 30%; P=0.14 | No serious risk | No serious inconsistency | No serious  indirectness | No serious imprecision | Undetected | Would not reduce effect | No | No | Low |
| Change in QRS duration | 30 | MD -19.6ms [-24.18, -15.02] | 83%; P <0.00001 | No serious risk | Serious inconsistency | No serious  indirectness | No serious imprecision | Undetected | Would not reduce effect | No | No | Very low |
| ACM | 16 | RR 0.87 [0.62, 1.22] | 48%; P=0.02 | No serious risk | No serious inconsistency | No serious  indirectness | Serious imprecision | Undetected | Would not reduce effect | No | No | Very low |
| HFH | 17 | RR 0.65 [0.49, 0.87] | 50%; P=0.01 | No serious risk | Serious inconsistency | No serious  indirectness | Serious imprecision | Undetected | Would not reduce effect | No | No | Very low |
| Echocardiographic non-response | 13 | RR 0.58 [0.41, 0.82] | 70%; P<0.0001 | No serious risk | Serious inconsistency | No serious  indirectness | Serious imprecision | Undetected | Would not reduce effect | No | No | Very low |
| Echocardiographic super-response | 6 | RR 1.86 [1.43, 2.43] | 34%; P=0.18 | No serious risk | No serious inconsistency | No serious  indirectness | No serious imprecision | NA | Would not reduce effect | No | No | Low |


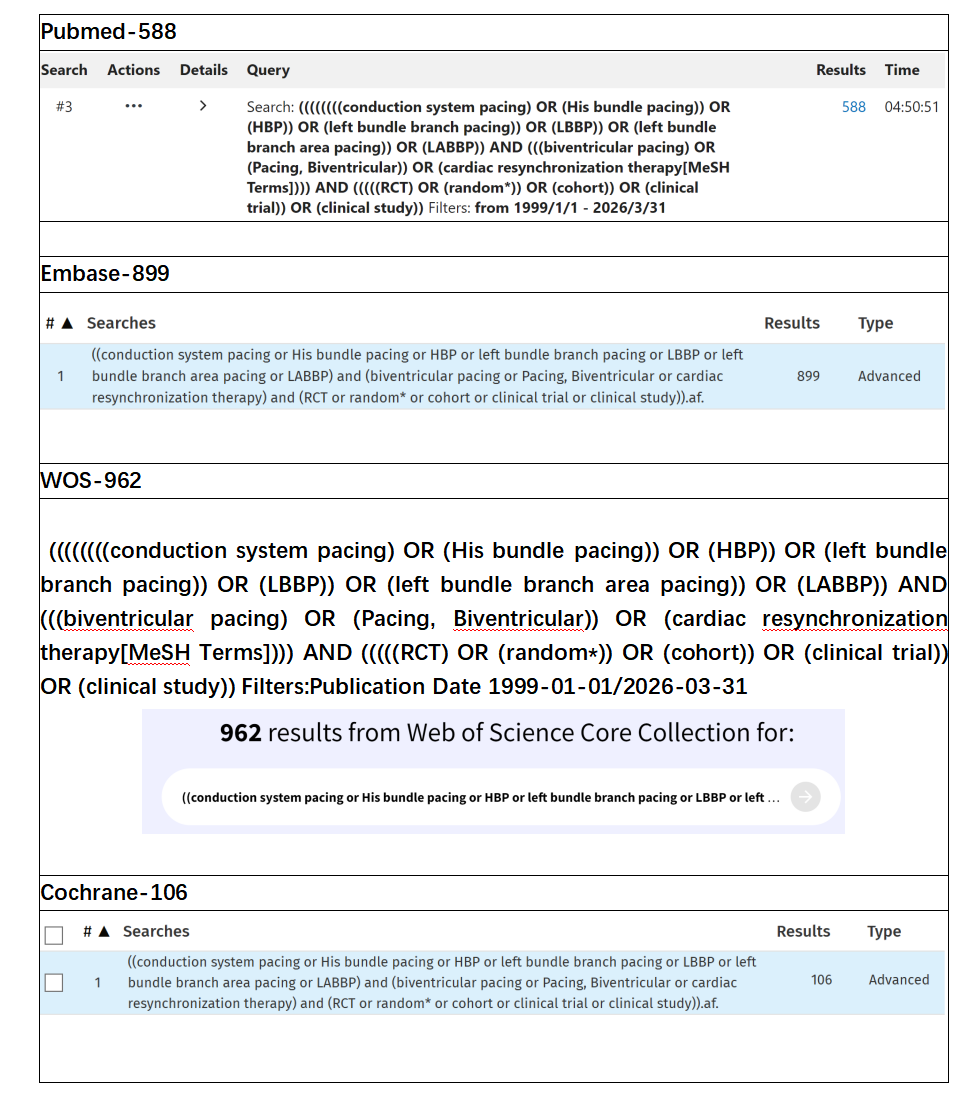


**Figure S1** Detailed search strategy.


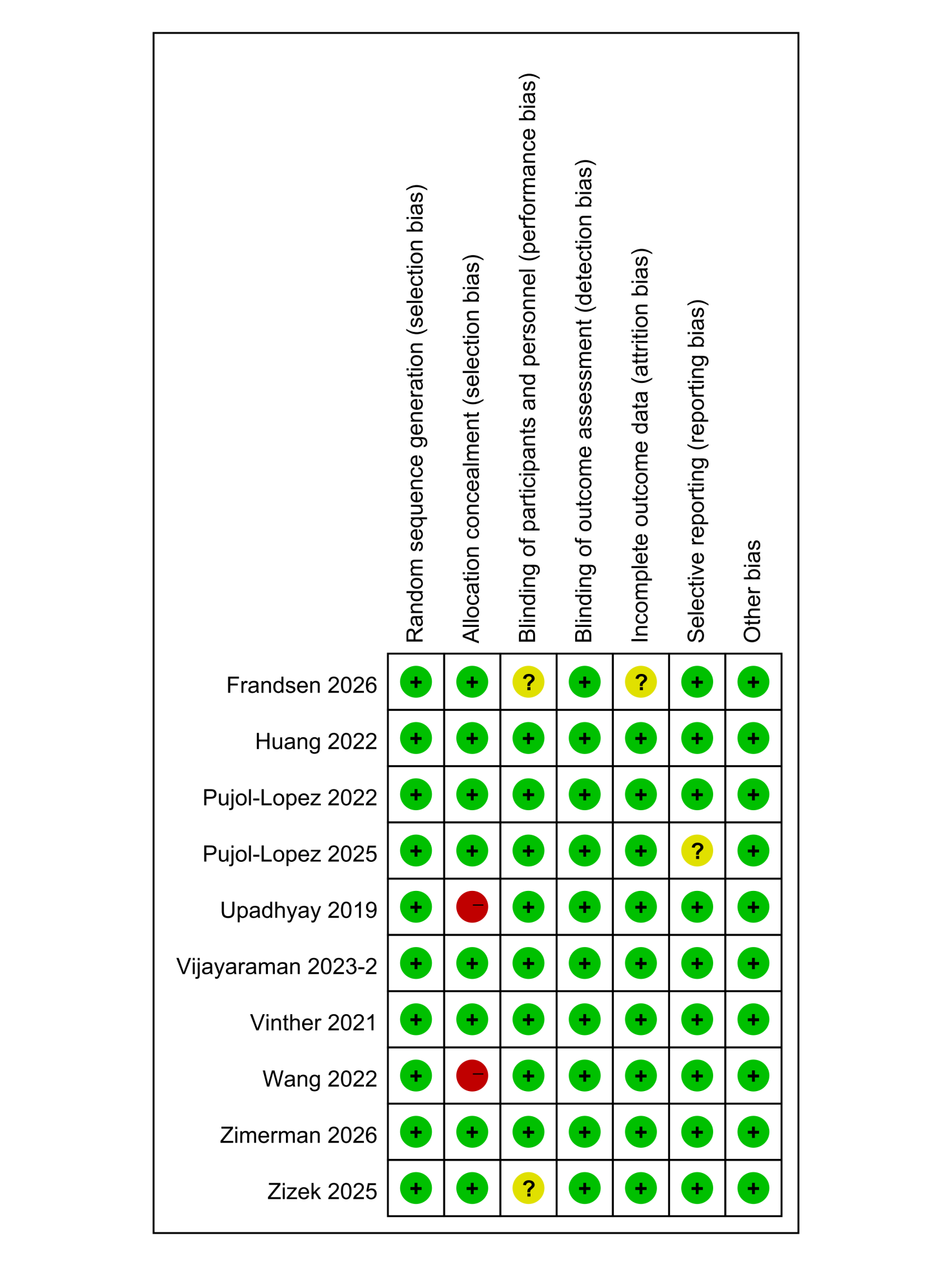


**Figure S2** Cochrane Handbook for Systematic Reviews of Interventions 5.1.0 was used to assess the RCTs’ quality.


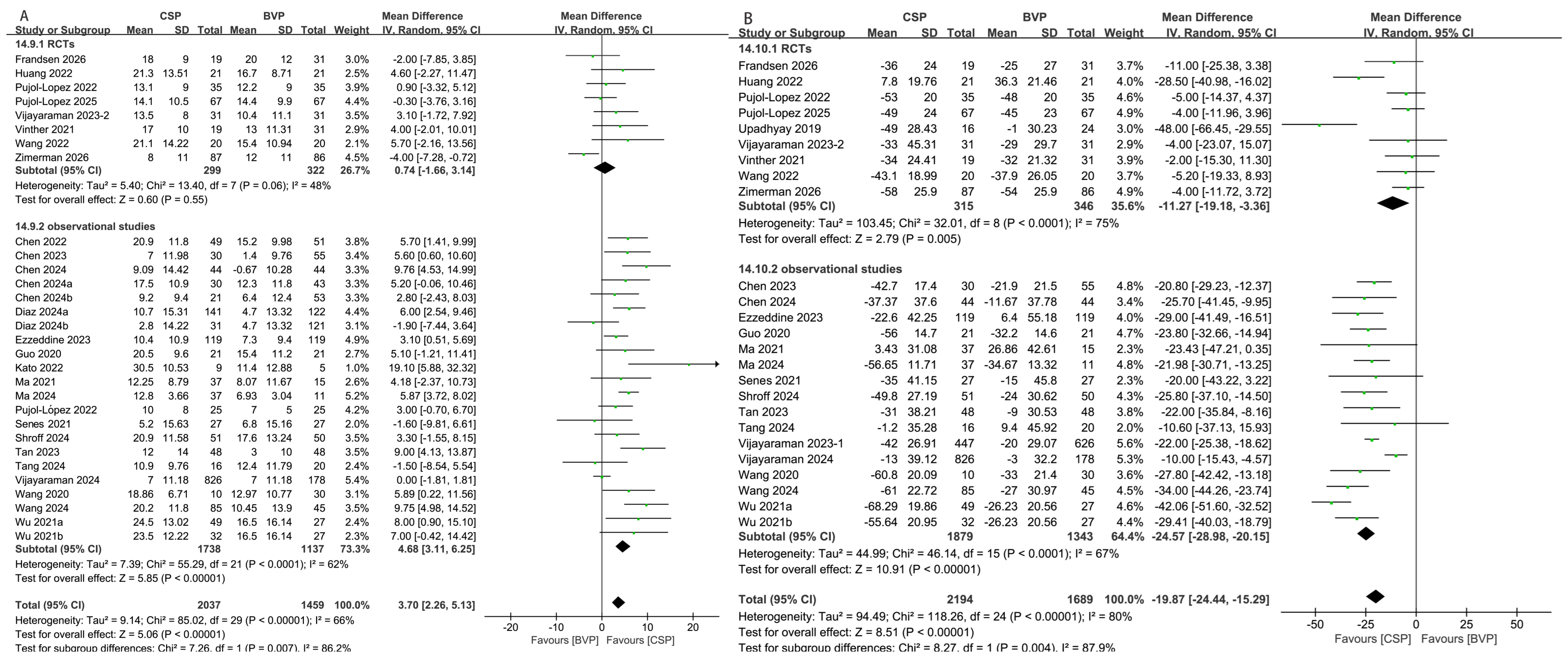


**Figure S3** Forest plots of outcomes after excluding studies with converted data (random-effects model). (A) change in LVEF and (B) change in QRS duration.


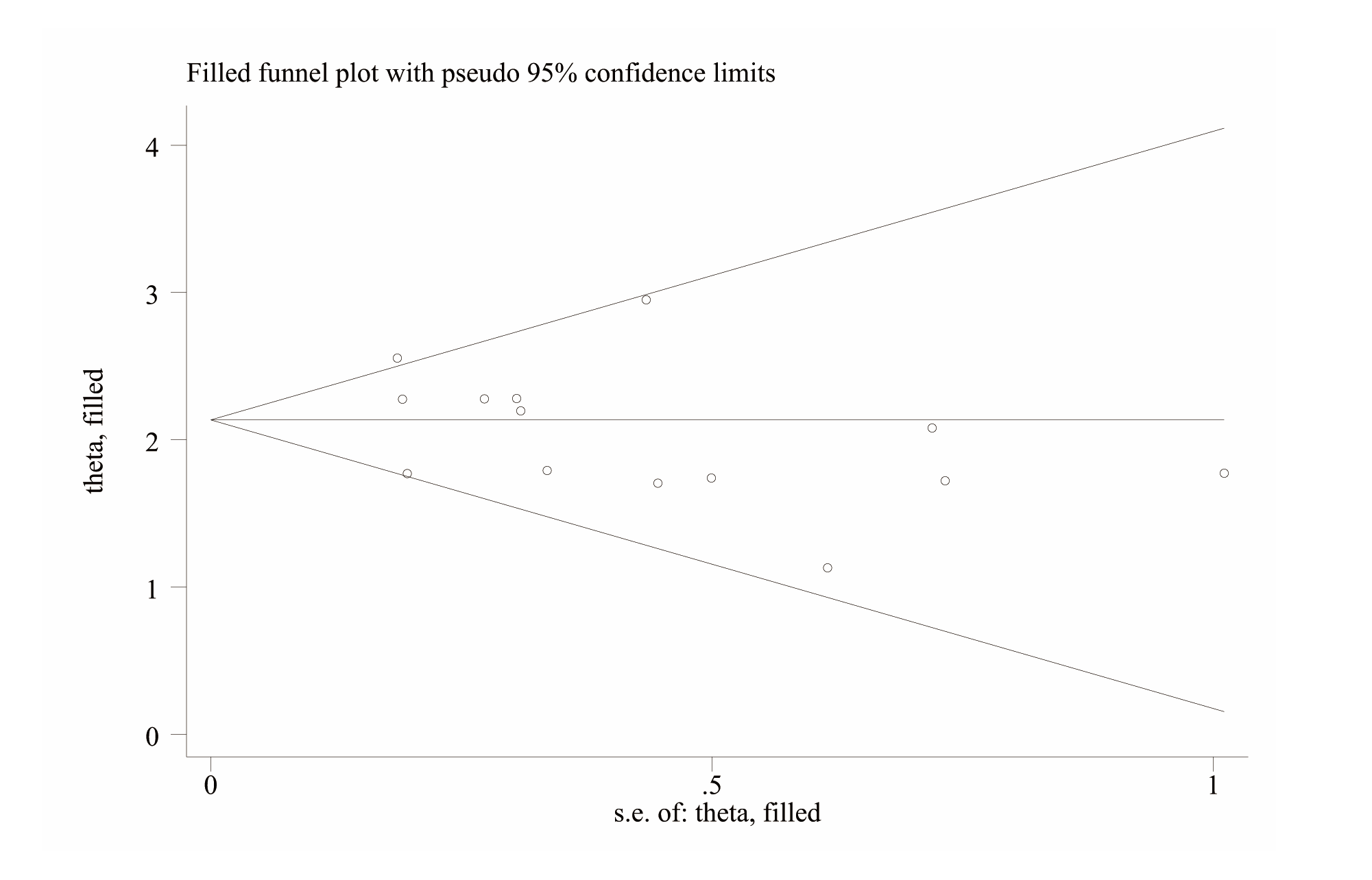


**Figure S4** Trim-and-fill funnel plot: change in LVEF.
